# A cynomolgus monkey with naturally occurring Parkinson's disease

**DOI:** 10.1093/nsr/nwaa292

**Published:** 2020-12-10

**Authors:** Hao Li, Ling-Yan Su, Lixin Yang, Min Li, Qianjin Liu, Zhenhui Li, Yan Hu, Hongwei Li, Shihao Wu, Wenchao Wang, Yingzhou Hu, Zhengbo Wang, Joshua D Rizak, Baihui Huang, Min Xu, Jing Wu, Long-Bao Lv, Christoph W Turck, Yong Yin, Yong-Gang Yao, Bing Su, Xintian Hu

**Affiliations:** Key Laboratory of Animal Models and Human Disease Mechanisms of the Chinese Academy of Sciences & Yunnan Province, Kunming Institute of Zoology, Chinese Academy of Sciences, China; Key Laboratory of Animal Models and Human Disease Mechanisms of the Chinese Academy of Sciences & Yunnan Province, Kunming Institute of Zoology, Chinese Academy of Sciences, China; State Key Laboratory of Genetic Resources and Evolution, Kunming Institute of Zoology, Chinese Academy of Sciences, China; Shenzhen Key Laboratory for Molecular Biology of Neural Development, Guangdong Provincial Key Laboratory of Brain Connectome and Behavior, CAS Key Laboratory of Brain Connectome and Manipulation, The Brain Cognition and Brain Disease Institute, Shenzhen Institutes of Advanced Technology, Chinese Academy of Sciences, China; Shenzhen-Hong Kong Institute of Brain Science, China; State Key Laboratory of Genetic Resources and Evolution, Kunming Institute of Zoology, Chinese Academy of Sciences, China; Key Laboratory of Animal Models and Human Disease Mechanisms of the Chinese Academy of Sciences & Yunnan Province, Kunming Institute of Zoology, Chinese Academy of Sciences, China; Kunming College of Life Science, University of Chinese Academy of Sciences, China; Key Laboratory of Animal Models and Human Disease Mechanisms of the Chinese Academy of Sciences & Yunnan Province, Kunming Institute of Zoology, Chinese Academy of Sciences, China; Kunming College of Life Science, University of Chinese Academy of Sciences, China; State Key Laboratory of Genetic Resources and Evolution, Kunming Institute of Zoology, Chinese Academy of Sciences, China; Kunming College of Life Science, University of Chinese Academy of Sciences, China; Key Laboratory of Animal Models and Human Disease Mechanisms of the Chinese Academy of Sciences & Yunnan Province, Kunming Institute of Zoology, Chinese Academy of Sciences, China; Key Laboratory of Animal Models and Human Disease Mechanisms of the Chinese Academy of Sciences & Yunnan Province, Kunming Institute of Zoology, Chinese Academy of Sciences, China; Institute of Neuroscience, CAS Key Laboratory of Primate Neurobiology, State Key Laboratory of Neuroscience, Chinese Academy of Sciences, China; Key Laboratory of Animal Models and Human Disease Mechanisms of the Chinese Academy of Sciences & Yunnan Province, Kunming Institute of Zoology, Chinese Academy of Sciences, China; Key Laboratory of Animal Models and Human Disease Mechanisms of the Chinese Academy of Sciences & Yunnan Province, Kunming Institute of Zoology, Chinese Academy of Sciences, China; Key Laboratory of Animal Models and Human Disease Mechanisms of the Chinese Academy of Sciences & Yunnan Province, Kunming Institute of Zoology, Chinese Academy of Sciences, China; Key Laboratory of Animal Models and Human Disease Mechanisms of the Chinese Academy of Sciences & Yunnan Province, Kunming Institute of Zoology, Chinese Academy of Sciences, China; Key Laboratory of Animal Models and Human Disease Mechanisms of the Chinese Academy of Sciences & Yunnan Province, Kunming Institute of Zoology, Chinese Academy of Sciences, China; Key Laboratory of Animal Models and Human Disease Mechanisms of the Chinese Academy of Sciences & Yunnan Province, Kunming Institute of Zoology, Chinese Academy of Sciences, China; Kunming College of Life Science, University of Chinese Academy of Sciences, China; Key Laboratory of Animal Models and Human Disease Mechanisms of the Chinese Academy of Sciences & Yunnan Province, Kunming Institute of Zoology, Chinese Academy of Sciences, China; National Resource Center for Non-Human Primates, Kunming Primate Research Center, and National Research Facility for Phenotypic & Genetic Analysis of Model Animals (Primate Facility), Kunming Institute of Zoology, Chinese Academy of Sciences, China; Key Laboratory of Animal Models and Human Disease Mechanisms of the Chinese Academy of Sciences & Yunnan Province, Kunming Institute of Zoology, Chinese Academy of Sciences, China; Max Planck Institute of Psychiatry, Department of Translational Research in Psychiatry, Germany; Department of Rehabilitation Medicine, the Second People's Hospital of Yunnan Province, China; Key Laboratory of Animal Models and Human Disease Mechanisms of the Chinese Academy of Sciences & Yunnan Province, Kunming Institute of Zoology, Chinese Academy of Sciences, China; Kunming College of Life Science, University of Chinese Academy of Sciences, China; Center for Excellence in Brain Science and Intelligence Technology, Chinese Academy of Sciences, China; National Resource Center for Non-Human Primates, Kunming Primate Research Center, and National Research Facility for Phenotypic & Genetic Analysis of Model Animals (Primate Facility), Kunming Institute of Zoology, Chinese Academy of Sciences, China; KIZ – CUHK Joint Laboratory of Bioresources and Molecular Research in Common Diseases, Kunming Institute of Zoology, Chinese Academy of Sciences, China; State Key Laboratory of Genetic Resources and Evolution, Kunming Institute of Zoology, Chinese Academy of Sciences, China; National Resource Center for Non-Human Primates, Kunming Primate Research Center, and National Research Facility for Phenotypic & Genetic Analysis of Model Animals (Primate Facility), Kunming Institute of Zoology, Chinese Academy of Sciences, China; Center for Excellence in Animal Evolution and Genetics, Chinese Academy of Sciences, China; Key Laboratory of Animal Models and Human Disease Mechanisms of the Chinese Academy of Sciences & Yunnan Province, Kunming Institute of Zoology, Chinese Academy of Sciences, China; Center for Excellence in Brain Science and Intelligence Technology, Chinese Academy of Sciences, China; National Resource Center for Non-Human Primates, Kunming Primate Research Center, and National Research Facility for Phenotypic & Genetic Analysis of Model Animals (Primate Facility), Kunming Institute of Zoology, Chinese Academy of Sciences, China

Evidence for the existence of monkeys with spontaneous Parkinson's disease (PD) has been lacking. Here, we screened macaque colonies at the Kunming Primate Research Center (Association for Assessment and Accreditation of Laboratory Animal Care (AAALAC) accredited), Kunming Institute of Zoology, Chinese Academy of Sciences, which has a large population of monkeys (>2400), to identify naturally occurring PD monkeys. Sixty cynomolgus macaques and rhesus macaques with no experimental experience, housed in single cages of the Kunming Primate Research Center, were selected as the subjects of a preliminary screen procedure to search for naturally occurring PD (Supplementary Fig. S1). The subjects’ age, gender and species information are listed in Supplementary Table S1.

Among the 60 monkeys, a 10-year-old male cynomolgus monkey (ID: #06103) showed severe PD symptoms. It had an overall PD score of over 15 (Fig. 1A), which was evaluated by part A of the improved version of the *Kurlan* scale [1]. The scale has been widely used in old world monkey PD symptoms quantification and its maximum score of part A is 20. The details of the rating scale are provided in Supplementary Table S2. According to UK Parkinson's Disease Society Brain Bank clinical diagnostic criteria [2], the core clinical symptoms used in PD diagnosis are bradykinesia, which is the essential criterion,

and at least one of two other symptoms, namely tremor and/or postural instability. A closer examination of the seven individual behavioral items’ scores that make up part A of the improved *Kurlan* scale showed that they were all above zero (Fig. 1B). Among them, there were all the behavioral symptoms employed in human PD diagnosis, including bradykinesia, tremor and postural instability, which was reflected in the balance, gait and gross motor skill disability of the monkey. Thus, we concluded that the cynomolgus monkey (ID: #06103) displayed all the testable diagnostic PD symptoms (Fig. 1A and B; Supplementary Video S1), completely fulfilled the clinical PD diagnostic criteria, and therefore was qualified as a monkey with Parkinsonism.

Following the classic PD symptoms assessments described above, a pharmacological validation was carried out. Compared with the symptom validation, which is a relatively rough quantification of the animal's daily behavior, a pharmacological validation is a better controlled and more PD specific quantitative test. In this test, the treatment of the monkey with a classic PD drug levodopa (L-dopa) resulted in positive responses with a significant total PD score reduction from 17.7 to 9.3 (*P* = 0.022, Fig. 1C; Supplementary Video S2). All seven individual items’ scores were reduced, with six out of seven being significant (Fig. 1D). Together, the above data

revealed very effective treatment responses and provided strong evidence for the pharmacological validation.

To confirm the L-dopa treatment effects, apomorphine (Apo), another classic PD drug which is an agonist of dopamine D1 receptor [2], was used. The monkey again displayed a positive treatment response with the total PD score significantly reduced from 15.3 to 10.3 (*P* = 0.023, Fig. 1E; Supplementary Video S3). The scores of three out of the seven items showed a significant reduction, another three showed a trend of reduction and one, gross motor skill, remained unchanged (Fig. 1F), also revealing effective treatment responses and providing a clear pharmacological validation. The same pharmacological tests were carried out on three control monkeys, which had an average total PD score 0.67 (the PD score range of normal monkeys is 0–4). All control animals showed no response to both PD drug treatments (Supplementary Fig. S2). Taken together, both the symptom assessment and pharmacological tests strongly suggested that we had successfully identified a monkey with Parkinsonism.

The key pathology hallmark of PD is severe loss of dopaminergic neurons and obvious morphological changes of the surviving dopaminergic neurons in the substantia nigra pars compacta (SNpc) [2,3]. Tyrosine hydroxylase (TH) immunostaining, which is a classic staining method for visualization of dopaminergic neurons, clearly showed that much fewer dopaminergic neurons were stained in the monkey with Parkinsonism compared with those in the death-condition-matched control (DCMC, ID: #071809) under lower resolution images (Fig. 1G and H). Under higher resolution images, obvious morphological changes of the surviving dopaminergic neurons, such as weaker-stained somata and fewer neurites could be observed (Fig. 1I and J). A quantitative analysis demonstrated an approximate 70% loss of dopaminergic neurons (Fig. 1K) in the SNpc of the monkey with Parkinsonism compared with the controls (the DCMC plus two age-matched controls; Supplementary Data). Collectively, the monkey with spontaneous Parkinsonism clearly possessed the most important PD pathology hallmark: severe loss of nigral dopaminergic neurons and obvious morphological changes.

**Figure 1.**
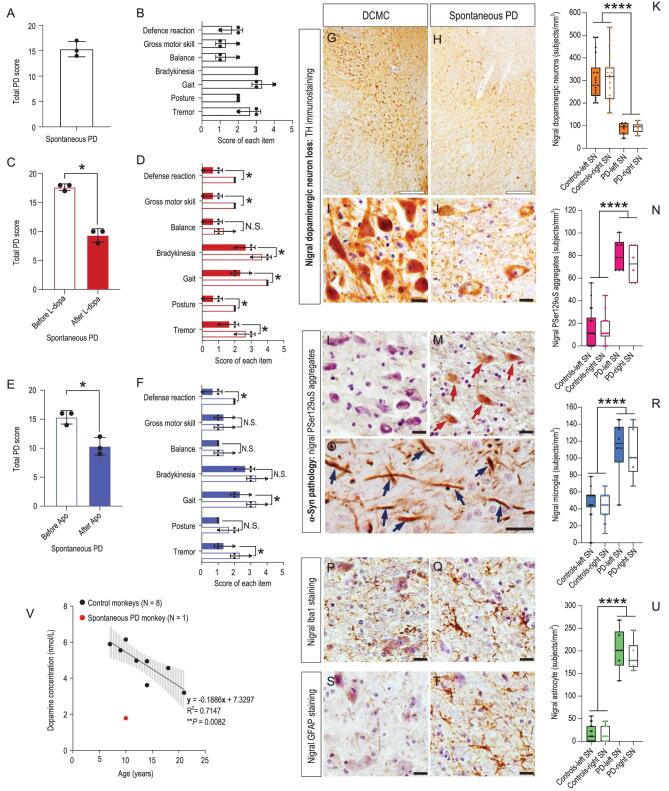
Classic Parkinsonian symptoms and pathologic hallmarks of the spontaneous PD monkey (#06103). (A) Quantified by the improved *Kurlan* scale, the monkey had a total PD score above 15 out of a maximum of 20, demonstrating severe PD symptoms. (B) The seven items’ scores that constitute the improved *Kurlan* scale were all above zero, revealing the monkey had all the diagnostic PD symptoms. The total PD score was the sum of the seven individual item scores. (C) The total PD score of the monkey was reduced significantly from 17.7 to 9.3 after a classic PD drug L-dopa treatment, indicating a significant positive response to the treatment. (D) The scores of the seven individual items were all reduced after the L-dopa treatment, and the reduction of six items out of the seven were significant. Both C and D provide strong evidence for pharmacological validation. (E) The total PD score of the monkey was reduced significantly from 15.3 to 10.3 after the apomorphine (Apo, an agonist of dopamine D1 receptor) treatment, indicating a significant improvement of PD symptoms by dopamine D1 receptor activation. (F) The scores of the seven individual items were all reduced after Apo treatment except for gross motor skill, and three out of the seven items were significant. These data further confirmed the above results from the L-dopa treatment. The data were quantified from three video clips of each experimental condition and presented as mean ± SD. Non-parametric statistics (Mann-Whitney test) were used (^*^, *P *< 0.05). (G and H) Under lower resolution images (4×), nigral TH immunostaining of the death-condition-matched control (DCMC, #071809) and spontaneous PD monkey indicated the overall nigral dopaminergic neuron loss of the spontaneous PD monkey: fewer nigral dopaminergic neurons that were lightly stained survived in the spontaneous PD monkey compared with the DCMC. (I and J) Under higher resolution images (40×), nigral TH immunostaining of the spontaneous PD monkey showed obviously less dopaminergic neurons with weakly stained somata and fewer neurites compared with that of the DCMC. (K) Quantitative analysis demonstrated that the nigral dopaminergic neuron number of the spontaneous PD monkey was about 70% less compared with that of the controls (^*^^*^^*^^*^, *P *< 0.001). (L and M) PSer129αS immunostaining of the SNs revealed that there were more PSer129αS aggregates (red arrows) in the spontaneous PD monkey compared with the DCMC. (N) The number of PSer129αS aggregates in the spontaneous PD monkey's SN was five times higher (^*^^*^^*^^*^, *P *< 0.001). (O) PSer129αS aggregates in the nigral neuron's processes were found in the spontaneous PD monkey (blue arrows). (P and Q) Iba1 immunostaining of the monkeys’ SNs revealed that there were more activated microglia in the spontaneous PD monkey compared with the DCMC. (R) The number of activated microglia in the spontaneous PD monkey's SN was 2.5 times larger than the controls (^*^^*^^*^^*^, *P *< 0.001). (S and T) GFAP immunostaining in the monkeys’ SNs revealed that there were more activated astrocytes in the spontaneous PD monkey compared with the DCMC. (U) The number of the activated astrocyte in the spontaneous PD monkey's SN was 12.5 times larger than the controls (^*^^*^^*^^*^, *P *< 0.001). Scale bars: hollow black: 200 μm; solid black: 20 μm. Data in K, N, R and U are median with minimum to maximum. Non-parametric statistics (Mann-Whitney test) was used. (V) Lower cerebrospinal fluid (CSF) dopamine level of the spontaneous PD monkey compared to the eight normal controls. The dopamine level was measured by a high-pressure liquid chromatography. Black dots represent dopamine concentrations of the eight normal control monkeys. The black line is the regression curve, and the gray shadow represents the 95% confidence interval of the control monkeys’ CSF dopamine concentration. The red dot represents the CSF dopamine concentration of the spontaneous PD monkey, which is located way outside the 95% confidence interval. ^*^, *P* < 0.05; ^*^^*^, *P* < 0.01 and ^*^^*^^*^^*^, *P* < 0.001.

Another important PD hallmark, Lewy body (LB) pathology, which appears in 77% to 95% PD patients according to different studies [3,4], is commonly illustrated by phosphorylated-serine129 α-synuclein (PSer129αS) immunostaining. Although classical Lewy bodies were not identified in this monkey, the α-synuclein pathology, which is also termed PSer129αS aggregates, was revealed by this staining. The

α-synuclein pathology is considered as an early stage of the Lewy pathology based on the following reasons: (i) aggregated α-synuclein is the major component of Lewy bodies and Lewy neurites [5], and about 90% of the α-synuclein aggregates consist of PSer129αS [6]; (ii) the LB formation is probably triggered by abnormal accumulation of PSer129αS in the cytoplasm [3,7]. Illustrated by staining with antibody *ab59264*, one of the most commonly used antibodies for PSer129αS immunostaining [7], a greater number of PSer129αS aggregates in the cytoplasm were found in the SNpc of the monkey with Parkinsonism compared with the DCMC (Fig. 1L and M). A quantitative analysis revealed that the number of PSer129αS aggregates was 5 times greater in the monkey with Parkinsonism than those in the controls (Fig. 1N). Moreover, some spindle-like or thread-like PSer129αS aggregates were also identified in the nigral neuron processes of the monkey with Parkinsonism; more evidence of the α-synuclein pathology (Fig. 1O). To further verify the specificity of the immunostaining results, a more specific monoclonal antibody (*ab51253*) was used to label the PSer129αS in the locus ceruleus (LC) of the monkey with Parkinsonism, which resulted in similar staining results (Supplementary Fig. S3). In summary, the

immunostaining results clearly illustrated that the PD candidate monkey had the α-synuclein pathologic changes, which is considered an early stage of the Lewy pathology.

A less emphasized but equally important pathology hallmark is the gliosis reported in PD patients [8]. It includes an increased number of microglia cells with enlarged somata and reduced but thickened neurites, and an increased number of astrocytes with expressed glial fibrillary acidic protein (GFAP). To explore the glia activation in the SN region of the monkey with Parkinsonism, Iba1 and GFAP antibodies were used to label the microglia and astrocytes, respectively. A large number of activated microglia (Fig. 1P and Q) revealed by enlarged somata (Supplementary Fig. S4) and astrocytes containing GFAP (Fig. 1S and T) were identified in SNpc of the monkey with Parkinsonism. Quantitative analyses showed a significant 2.5-fold increase of microglia (Fig. 1R) and a 12.5-fold increase of astrocyte numbers (Fig. 1U) in this monkey. Moreover, consistent with what is observed in PD patients [9], a significantly lower cerebrospinal fluid (CSF) dopamine level was identified in the monkey with Parkinsonism (Fig. 1V, red dot). Taken together, the identified monkey (ID: #06103) displayed all the classic PD pathology hallmarks, and therefore was fully qualified as a spontaneous PD monkey in terms of pathological changes, in addition to the behavioral and pharmacological validations.

This PD monkey was 10 years old, which corresponds to a human age of around 30–40 [10]. Since most early-onset PD patients have genetic contributions [2], we carried out a sequencing analysis of 11 known PD risk genes and delineated four missense mutations in *LRRK2* and *ATP13A2* in this monkey (Supplementary Figs S5 and 6). Follow up functional studies suggested that the *LRRK2* mutations were likely causal for PD pathology (Supplementary Figs S7 and 8).

Collectively, four lines of evidence, including typical PD clinical symptoms, pharmacological responses, pathology hallmarks and genetic mutations, strongly suggested that we had identified a cynomolgus monkey with spontaneous PD. This indicates that PD is not a uniquely human disease and, therefore, as close relatives to humans, monkeys are ideal candidates to develop genuine ‘animal versions of PD’, in which the etiology and pathogenesis are evolutionally conserved. Furthermore, it allows one to compare similarities and differences of PD development between species and to understand PD pathogenesis from an evolutionary point of view.

## Supplementary Material

nwaa292_Supplemental_File
