# Supplementary material for: A cynomolgus monkey with naturally occurring Parkinson's disease
Source: Natl Sci Rev. 2020 Dec 10;8(3):nwaa292. doi: 10.1093/nsr/nwaa292 (PMC8288342; doi:10.1093/nsr/nwaa292)
Supplement: nwaa292_Supplemental_File [file nwaa292_supplemental_file.zip › Supplementary Data-R1.pdf]

Supplementary Data

A cynomolgus monkey with naturally occurring Parkinson's disease

Hao Li, Ling-Yan Su, Lixin Yang, Min Li, Qianjin Liu, Zhenhui Li, Yan Hu, Hongwei Li, Shihao Wu, Wenchao Wang, Yingzhou Hu, Zhengbo Wang, Joshua D. Rizak, Baihui Huang, Min Xu, Jing Wu, Long-Bao Lv, Christoph W. Turck*, Yong Yin*, Yong-Gang Yao*, Bing Su*, and Xintian Hu*

***Corresponding authors (Emails):**
turck@psych.mpg.de;
yinyong@ynshhyy.com;
yaoyg@mail.kiz.ac.cn;
sub@mail.kiz.ac.cn;
xthu@mail.kiz.ac.cn.

<u>This PDF document contains:</u>

Supplementary Methods
Supplementary Results
Supplementary Figures S1-S10
Supplementary Tables S1-S4
Supplementary References 1-56

Supplementary Methods

Animals and ethics

The experimental protocols and animal welfare were approved by the Ethics Committee of Kunming Primate Research Center (AAALAC accredited), Kunming Institute of Zoology, Chinese Academy of Sciences. All animals involved in this study were treated also in accordance with the National Institute of Health (U.S.A.) Guide for the Care and Use of Laboratory Animals [*8th edition. Washington (DC): National Academies Press; 2011*] and the ARRIVE guidelines for reporting animal research [1]. Here, 34 rhesus monkeys (*M. mulatta*) and 26 cynomolgus monkeys (*M. fascicularis*) were selected as the primary subjects for this project based on the following terms, 1) age: 7-30 years old; 2) general status: healthy and without any prior experimental experience. The detailed information of these 60 monkeys is listed in Table S1, and best efforts were made to minimize the suffering of the monkeys involved in this study.

Selection and validation procedures for the PD monkey candidates

The first criterion for selection was the appearance of one or more Parkinsonian symptoms, including bradykinesia, tremor, and postural instability. After the first screen, a 10-year old male cynomolgus monkey (ID: #06103) was identified as a candidate. Next, four quantitative assessments were carried out to validate the monkey was indeed a PD case.

1) PD behavioral symptoms validation: PD symptoms of the candidate monkey and three control monkeys were quantified by the well-established *Kurlan* scale (improved version) [2], a widely used scale to quantify PD symptoms in old world monkeys [3].

2) Pharmacological validation: the PD monkey candidate and three control monkeys were treated with levodopa (L-dopa) and apomorphine (Apo) to evaluate their responses to the classic PD drugs used clinically for PD patients. Improved responses resemble those observed in PD patients are in support of the monkey suffering from PD.

3) Pathologic validation: the pathology hallmarks of PD include severe nigral dopaminergic neuron loss, α-synuclein pathology, glial cell activation and low dopamine concentration in the brain. This included nigral tyrosine hydroxylase (TH) immunostaining to illustrate dopaminergic neurons, phosphorylated Ser129 α-synuclein immunostaining to illustrate α-synuclein pathology, and Iba1 and

GFAP immunostaining to show microglia and astrocyte activation, respectively. The dopamine concentration in the cerebrospinal fluid (CSF) of the PD candidate monkey was measured by a high-performance liquid chromatography (HPLC) and then compared with those of eight normal controls.

4) Genetic analysis and the related functional assays: to identify PD-related mutations, a total of eleven reported PD risk / causal genes were sequenced for the PD candidate monkey and compared with our genetic database of 1,585 normal cynomolgus monkeys. Afterwards, the functional consequences of the identified mutation(s) were investigated at the cellular level, and RNA-seq was performed to reconstruct the differentially expressed genes and affected pathways.

Detailed information of the four validations

1) PD symptoms validation

To identify and quantify the Parkinsonian symptoms of the PD monkey candidate (ID: #06103), its behavior was video recorded according to a protocol adopted from previous studies [2, 4, 5]. Free moving behavior of the monkey in a single cage was recorded without external disturbance for 1 hour by a digital camera (Sony HDR-XR260, Japan) located 1 meter in front of the cage from 15:00 pm to 16:00 pm. After the recording, the 1-hour video was divided into 3 episodes of 20 minutes each by the experimenter, creating 3 video clips for further behavioral assessment.

The video analysis protocol was adopted from previous studies as well [2, 4, 5]. Two experienced observers were involved in the analysis. The 3 video clips were randomly labeled by the experimenter with numbers from 1 to 3. Blind to the conditions of the monkey, the two observers were assigned the video clips to score independently using the well-established *Kurlan* scale (improved version) [2], which has been widely accepted as a standard scale for PD research on old world monkeys [3].

There are 4 parts in the improved *Kurlan* scale: part A: Parkinsonian features; part B: drug-related side effects; part C: overall level of activity; part D: clinical staging. Only parts A and D were used in this study. The symptom of the PD monkey was marked by part A of this scale [2], which included seven items: tremor, posture, gait, bradykinesia, balance, gross motor skill, and defense reaction. Each item score was evaluated and assigned by the severity of the symptom (*Tremor: 0~3; Posture: 0~2; Gait: 0~4; Bradykinesia: 0~4; Balance: 0~2; Gross motor skill: 0~3; Defense reaction: 0~2*),

and the total PD score was the sum of the scores of the seven items. The total score maximum is 20, and the range of the normal score is about 0-4. For more details, please refer to Table S2. If there was no obvious scoring difference (less than two points) between the two observers, the data was combined to create figures and perform statistics analysis.

The PD monkey candidate's clinical stage, as assessed by part D of the scale, was determined as the most severe stage V according to following three criteria: **1)** bilateral Parkinsonism, obvious by no rotational behavior following apomorphine (Apo) treatment. Usually, monkeys will show obvious rotational behavior after Apo administration if they are hemi-Parkinsonism [4, 6]. Moreover, there were similar severe dopaminergic neuron losses in both the left and right SNs ($P = 0.801$, *t*-test); **2)** required assistance to maintain adequate nutrition, which was obvious by difficulties that the monkey had during eating (data not shown); **3)** frequent face-down posture (please refer to Supplementary Videos 1-3).

2) Pharmacological validation

Levodopa (L-dopa) and apomorphine (Apo) are two classic PD treatment drugs and also standard agents in PD animal models' pharmacological validation [7, 8]. The L-dopa treatment effect was evaluated by comparing the monkey's PD score marked by the improved *Kurlan* scale mentioned above before and after the L-dopa administration. Briefly, behavior of the PD monkey candidate was recorded for 1 hour from 15:00 pm to 16:00 pm, and then the PD monkey candidate was intramuscularly injected (*i.m.*) with 2 mL L-dopa (30 mg/kg, V-900425-100MG, Sigma, U.S.A.). After 15 minutes, the behavior of the PD monkey candidate was recorded again for 1 hour from 16:20 pm to 17:20 pm. Drug effects of L-dopa wash out usually takes 9 hours.

Next day, the Apo treatment effect was evaluated in the same way as the L-dopa treatment, except that the dosage was 1 mg/mL, 2 mL, *i.m.* (A4393-100MG, Sigma, U.S.A.).

After these tests, 4 videos of 1 hour each were obtained from the PD monkey candidate. Each individual video was divided into 3 episodes of 20 minutes by the experimenter, creating 12 video clips of 20 minutes each, which were randomly labeled by the experimenter with the numbers from 1 to 12. Conditions of the clips were blind to the two observers who analyzed the videos in the same way as the PD symptoms validation.

3) Pathologic validation

a) Control monkeys' information and brain tissue treatment

The PD monkey candidate was weak and thin when we first noticed it. It was carefully cared for by veterinarians after that. Not long after the behavioral tests were finished, the monkey died during an unexpected cold night. The body was examined next morning and, thus, the postmortem interval was less than 12 hours. No obvious abnormity was observed during the autopsy, leading us to conclude that the death was probably due to hypothermia.

To verify the pathology hallmarks of the PD monkey candidate, valid controls were needed. There were two kinds of controls in this study. The first was a death-condition-matched control (DCMC), since the brain tissue of the PD monkey candidate was acquired after its sudden death without perfusion. To exclude potential pathology changes that might be caused by the non-perfusion process, brain tissue from an age-matched male cynomolgus monkey (ID: #071809), who also died of cold weather, was acquired within 12 hours without perfusion just like the PD monkey candidate, and was used as a DCMC for PD pathology study. The brain tissues of the two monkeys were all fixed in 4% paraformaldehyde-0.01 M PBS for two weeks, and then they were dehydrated in 20% and 30% sucrose solutions.

The second kind of control was age-matched normal monkeys. To increase the statistic power, two more age-matched monkeys (ID: #071725, and #05103) were first intramuscularly administrated with ketamine (10 mg/kg, 0.05 g/mL, *i.m.*, Zhongmu, Taizhou, China) and then euthanized with pentobarbital sodium (40 mg/mL, 20 mg/kg, *i.m.* Fluka, Germany) for perfusion. After the perfusion, their brains were processed in the same way as the two monkeys of sudden death mentioned above.

b) Determination of the anterior-posterior length (AP length) of the substantia nigra (SN) in monkey brain

The most important brain area involved in PD pathology is the substantia nigra (SN), a paired flat shaped structure located deeply in the narrow mesencephalon [4, 9]. It is necessary to determine the anterior-posterior length (AP length) of the SN before the sectioning. The length of cynomolgus monkey's SN was determined by referring to "*The Rhesus Monkey Brain in Stereotaxic Coordinates, George Praxinos et al., Academic Press, 1999*" and then verified by a set of MRI scanning data of cynomolgus monkeys from a 3T MRI facility (United Imaging, Shanghai, China) in Kunming Institute of Zoology, Chinese Academy of Sciences.

First, according to the monkey brain atlas, the SN of macaque monkey brain is located at -9.90 mm to -17.78 mm AP from the bregma. As a result, its AP length is 7.88 mm (17.78 - 9.90 = 7.88 mm). Second, using the MRI data, the AP length of the cynomolgus monkeys' SNs was measured with a range from 6.7-7.8 mm. To ensure inclusion of the monkey's whole SN, the total AP length of the cynomolgus monkey's SN was determined as 8.0 mm.

c) Cutting brain sections for immunostaining

The total AP length of the monkey's SN was determined as 8.0 mm by the procedure mentioned above. After this, the SN was cut into coronal sections with a thickness of 20 μm. The interval between every two sections was 60 μm, and, as a result, about 100 sections were obtained from each monkey's SN. All the monkeys' SNs were cut in the same way by a freezing microtome (Leica, CM1850UV-1-1, Germany) at -24 °C.

Next, we needed to pick out the optimum sections of the cut SN for immunostaining. The anatomical criteria for selecting the optimal portion of SN for pathological study was established by Chu *et al.* [10-12], Gundersen *et al.* [13] and also by Dickson *et al.* [9]. The criteria are to select the portion of the SN that overlaps with the third cranial nerve (3n) rootlets. The SN is in the ventral midbrain and defined ventrally by the cerebral peduncle and medially by the 3n rootlets in a coronal section [9, 11]. According to the monkey's atlas, the total AP length of the SN that overlaps with the 3n rootlets is 2.25 mm (-10.80 mm to -13.05 mm AP from bregma), near 30% of the whole SN (2.25/8 = 28.13%), and, according to the criteria, the optimum sections of a SN for staining were around 30 coronal sections, ranging from the 11th section to the 40th section out of the 100 sections (Fig. S9). As for the three controls, their SNs were sectioned and selected in the same way.

d) Staining

The 30 optimum sections of SN were divided into 5 sets for TH, PSer129αS, Iba1, GFAP immunostaining, and hematoxylin & eosin (H & E) staining, respectively. A total of six sections were picked out from every 5 sections among the 30 to form a set for the staining (Fig. S9).

For TH staining, a standard protocol was used [5, 11]: the sections were treated with 3% H_2O_2 (5 min, Maixin, Fuzhou, China), 3% triton X-100 (4 min, Solarbio, Beijing, China), 10% goat serum (15 min, Maixin, Fuzhou, China) and then incubated with rabbit anti-TH antibody (1: 1000, AB152, Millipore, U.S.A.) overnight at 4 °C. On the second day, the sections were incubated with anti-

rabbit/mouse secondary antibodies kit (PV-9000, ZSGB-BIO, Beijing, China) for 30 min at 37 °C. After that, the sections were incubated with DAB (DAB-1031Kit, 20 ×, Maixin, Fuzhou, China) for 2 min and counterstained with hematoxylin for 1 min.

For the PSer129αS staining, a previously published protocol was used [14-16]. The sections were treated with or without 50 μg/mL proteinase K (Aladdin, Shanghai, China) in 10 mM Tris-HCl, pH = 7.8, 100 mM NaCl, 0.1 % Tween-20 (Bio-Rad, U.S.A.) at 37 °C for 30 min. After that, sections were treated with 3% H_2O_2 (5 min, Maixin, Fuzhou, China), 3% triton X-100 (4 min, Solarbio, Beijing, China), 10% goat serum (15 min, Maixin, Fuzhou, China) and incubated with a commonly used [14, 16-21] rabbit anti-PSer129αS polyclonal antibody (1: 100, ab59264, Abcam, U.K.) overnight at 4 °C. On the second day, the sections were incubated with anti-rabbit/mouse secondary antibodies kit (PV-9000, ZSGB-BIO, Beijing, China) for 30 min at 37 °C. After that, sections were incubated with DAB (DAB-1031Kit, 20 ×, Maixin, Fuzhou, China) for 2 min and counterstained with hematoxylin for 1 min. To test the specificity of PSer129αS staining, we used another classic rabbit monoclonal antibody for labeling PSer129αS aggregates (ab51253, Abcam, U.K.) [12, 22]. The staining protocol was the same as described above but the antibody was changed to ab51253 (1:1000, Abcam, U.K.).

For the glial cells staining, astrocytes and microglia were stained in a standard way [23]. The sections were treated with 3% H_2O_2 (5 min, Maixin, Fuzhou, China), 3% triton X-100 (4 min, Solarbio, Beijing, China), and 10% goat serum (15 min, Maixin, Fuzhou, China). For the astrocyte staining, the sections were incubated with mouse anti-GFAP antibody (1:1000, SMI21-R, Biolegend, U.S.A.) overnight at 4 °C. For microglia staining, the sections were incubated with rabbit anti-Iba1 antibody (1:500, 019-19741, Wako, Japan) overnight at 4 °C. After that, the sections were incubated with anti-rabbit/mouse secondary antibodies kit (PV-9000, ZSGB-BIO, Beijing, China) for 30 min at 37 °C. After that, sections were incubated with DAB (DAB-1031Kit, 20 ×, Maixin, Fuzhou, China) for 2 min and counterstained with hematoxylin for 1 min.

Hematoxylin and eosin (H & E) staining was used to identify the nigral pigmented cells (dopaminergic neurons) by the darkly stained hematoxylin and for staining the classical LBs with acidophilic inclusions. It was performed following the H & E kits instructions (G1120, Solarbio, Beijing, China). The brain sections were stained in the hematoxylin solution for 1 min and then washed with water. The sections were then treated with 1% hydrochloric acid-alcohol solution for 10

sec, washed back to blue and then stained in the eosin solution for 10 sec. After that, the sections were washed clean with water.

After the staining, all the sections were dehydrated with an alcohol gradient and xylene. Neutral balsam was used to cover the sections and digital pictures were taken with a microscope (Olympus, CX41; camera: Olympus DP25; software: CellSens Entry 1.4.1; Japan). For the three controls, the sections were stained in the same way.

Immunofluorescence staining was used to test the role of mutant LRRK2 *in vitro*. Cultured U251 cells stably expressing different LRRK2 mutations were fixed and stained with monoclonal antibody against serine 129-phosphorylated α-synuclein (1:1000, ab51253, Abcam, U.K.). Immunoreactivity was detected with a FITC-conjugated secondary antibody (1:500, A-21207, invitrogen, U.S.A.) and nuclei were counterstained with DAPI and the slides were visualized under an Olympus FluoView™ 1000 confocal microscope (Olympus, U.S.A.).

e) Stereological (cell) counting

After the immunostainings, stereological counting was carried out to quantify the pathological changes. The protocols were from previous reports [11, 13, 24, 25]. A typical immunostained section was screened under a 4 × objective (3.5 mm × 2.6 mm), a 20 × objective (690.8 μm × 518.1 μm) and a 40 × objective (345.4 μm × 259 μm) to locate the substantia nigra pars compacta (SNpc) for cell counting, because most nigral dopaminergic neurons are located in this area [9, 26]. The location of the counting frame on a section of the controls or the PD monkey candidate was at the same part of the SNpc, which was in the middle of the long axis of the SNpc (Figs. S10A-B, blue frame). This location was determined by both the anatomical characteristics of the SNpc (ellipsoid shaped and defined ventrally by the cerebral peduncle and medially by the third cranial nerve) and the cellular properties of the SNpc: pigmented cells (H & E staining in Fig. S10C), spindle-shaped cells (TH immunostaining in Fig. S10D) and their even distribution in the area (Fig. S10B).

The number of nigral dopaminergic neurons (TH^+ cells) in the area of the counting frame was counted by ImageJ software (National Institutes of Health, Bethesda, Maryland, USA) under the 40 × objective to ensure correct reorganization of the morphology of an individual cell (Fig. S10B, blue frame and zoomed in Fig. S10D). To quantify the nigral dopaminergic neuron loss in the PD monkey candidate, two additional age-matched normal control monkeys (ID: #071725 and #05103) were

interrogated with the DCMC to increase the statistical power. There were no significant differences in the SNpc dopaminergic neuronal count among the three control monkeys ($P = 0.986$, Chi-square test), and also no significant differences in the left and right side of SNpc of the three control monkeys ($P = 1.00$, *t*-test). Therefore, the averaged density of cells (subjects/mm^2) obtained from the left and right SNs of the controls (in total 18 sections containing the left and right SNs) were pooled together for statistics. For the PD monkey candidate, the dopaminergic neuron counting from both sides of the SNs (in total 6 sections containing the left and right SNs) was pooled together for no significant difference ($P = 0.801$, *t*-test), and we found the averaged density of nigral dopaminergic neuron of the PD monkey candidate was close to that of PD patients [27].

To verify the reliability of the counting method, the total number of dopaminergic neurons in SNpc was estimated by a classic analysis method used in Garcia *et al.* [23] and Gundersen *et al.* [13]. The averaged total number of neurons in the SNpc of the three controls was around 13900, consistent with previous studies (around 14500) [11, 12], suggesting the counting was reliable. The stereological counting of the nigral PSer129αS aggregates, microglia cells (nigral $Iba1^+$ cells) and astrocytes (nigral $GFAP^+$ cell) were carried out in the same way.

f) Dopamine concentration measurement

The monkeys involved in this measurement (Table S1) were anesthetized with ketamine (10 mg/kg, 0.05 g/mL, i.m., Zhongmu, Taizhou, China). Approximately 1.5 mL CSF (divided into three 0.5 mL samples) of each monkey was collected with a lumbar puncture using a 22-gauge needle by an experienced technician. The collected CSF samples were immediately frozen in liquid nitrogen and stored at −80°C. Before the high-pressure liquid chromatography (HPLC) measurement, the CSF samples were thawed and centrifuged at 20000g for 15 min at 4 ºC. Then, the dopamine (DA) concentration of each monkey was measured by HPLC (Antec 110, Holland) using an ALF-115 & ALF-105 column (Antec, Holland).

4) Genetic analysis and the related functional assays

a) Gene sequencing

Approximately 2 mL blood was collected from each of 1,585 cynomolgus monkeys for whole genome DNA extractions. Eleven PD risk / causal genes (*ATP13A2, FBXO7, GBA, GIGYF2, HTRA2, LRRK2, MAPT, PINK1, PLA2G6, SNCA, and UCHL1*) were selected for targeted

sequencing based on the severity of mutations previously published in PD exome sequencing studies [26, 28, 29]. An improved algorithm, MIPgen [30] was used to design molecular inversion probes (MIPs), which are single stranded DNA molecules containing two regions complementary to regions in the targeted DNA [31-33]. For the 11 targeted genes, the final probe designs included 359 smMIPs. Oligonucleotides were obtained from Integrated DNA Technologies (IDT, Coralville, IA, USA). MIPs were pooled by gene. The MIPs, 100 ng genomic DNA, 0.32 mM dNTPs, 0.32 μL Hemo KlenTaq (New England Biolabs, Inc., Ipswich, MA, USA) and 1 unit of Ampligase (Epicentre) were mixed with 1 × Ampligase buffer (Epicentre, Madison, WI, USA) in a PCR tube with a total reaction volume of 25 μL. Gap filling and ligation were also performed in the same PCR tube. The PCR tubes were incubated at 95°C for 10 min and then 60°C for 22 h. Subsequently, 2 μL exonuclease mix was added into the same tube and incubated at 37°C for 45 min and then 95°C for 2 min. The captured DNA was amplified as previously reported [34] and 5 μL amplified products were pooled together and purified with 0.9 × AMPure XP beads (Beckman Coulter, Brea, CA, USA) according to standard protocol. Then gel visualization with 2% agarose gel and quantification with the Qubit dsDNA HS Assay (Life Technologies, Grand Island, NY, USA) were performed. Approximately 384 individuals were combined as the final pool for sequencing in one lane of an Illumina HiSeq 3000. The sequences were mapped to RheMac3 (http://genome.ucsc.edu/cgi-bin/hgGateway?db=rheMac3) using BWA-MEM [35]. For quality control, read pairs with incorrect pairs and insert sizes were removed after mapping, leaving only the reads with the highest quality scores. FreeBays [30] was applied for variant calling and SeattleSeq138 (https://snp.gs.washington.edu/SeattleSeqAnnotation137) for variants annotation. For the validation of variants, 300 bp primers were designed using BatchPrimer3 (https://wheat.pw.usda.gov/demos/BatchPrimer3). PCR was performed with a standard program in 25 μL reaction volume. The PCR products were mixed and purified with 0.9 × AMPure XP beads and the product was sent for Sanger sequencing.

The MIP sequencing information for PD-risk / causal genes in the spontaneous PD monkey and Combined Annotation Dependent Depletion (CADD) score [36, 37] is listed in Table S3.

b) Construction of U251 cells with stable LRRK2 mutant expression

We performed cellular assays to characterize the potential effect of the LRRK2 mutants p.E2381G,

p.N1506Y and p.I835L. We generated three mutants (p.E2381G, p.N1506Y and p.I835L) based on the wild type (WT) LRRK2-pCMV-c-Myc vector, constructed in our previous study [38], by using site-directed mutagenesis PCR methods. All mutants were verified by sequencing. All primer information is shown in Table S4.

To generate a stable and inducible cell line expressing WT or mutant LRRK2 upon doxycycline (Dox, Sigma, St Louis, MO, U.S.A.) induction, we used Lenti-X Tet-On Advanced Inducible Expression System (Clontech) following our previously reported method [38]. In brief, WT or mutant LRRK2 was subcloned into pLVX-tight-puro (Clontech) by using two sets of primers to introduce restriction endonuclease sites (*Not*I and *Mlu*I) and the c-Myc tag (Table S4). The response lentivirus system was composed of WT or mutant LRRK2 constructs, packaging plasmid psPAX2 (12260, Addgene, U.K.) and envelope plasmid PMD2.G (12259, Addgene, U.K.), while the regulator lentivirus system was composed of PLVX-Tet-On-Advanced vector, psPAX2 and PMD2.G. The HEK293T cells were introduced from the Kunming Cell Bank, Kunming Institute of Zoology, and were cultured in DMEM (11965, Gibco, U.S.A.) containing 10% heat inactivated FBS. Cells were transfected with LRRK2 or Advance vector, packaging plasmid psPAX2 and envelop plasmid PMD2.G with a ratio of 3:2:1. The lentivirus was collected from cell supernatant at 48 h after transfection. The U251 cells were maintained in RPMI 1640 supplemented with 10% FBS (11875, Gibco, U.S.A.). The lentivirus supernatant was produced from HEK293T cells and was used to infect U251 cells with the ratio of 4:1 for the response lentivirus and the regulator lentivirus. Cells were selected in growth medium with 500 μg/mL G418 and 2 μg/mL puromycin for 2 weeks. Control U251 cells, U251 cells with pLVX-tight-puro (Clontech) and U251 cells with LRRK2 were cultured in a 6-well plate with 2 μg/mL doxycycline (Sigma) for 48 h to induce LRRK2 protein expression.

c) Western blotting

The U251 cells stably expressing different LRRK2 mutants were harvested for Western blot and RNA-seq. Total cellular protein was obtained by using cell lysis buffer (P0013, Beyotime, China) and the protein concentration was determined by BCA Protein Assay Kit (P0012, Beyotime, China). Western blots were performed using the method described in our previous studies [39, 40]. In brief, 30 μg protein was separated by 10–15% sodium dodecyl sulfate-polyacrylamide gel electrophoresis, transferred to a polyvinylidene difluoride membrane (Bio-Rad, Hercules, CA, U.S.A.) and the

membrane was blocked with 5% (w/v) skim milk for 2 h at room temperature. The membrane was then incubated with the respective primary antibody against c-Myc (1:1000, C3956, Sigma, U.S.A.), LRRK2 (1:1000, ab133518, Abcam, U.K.), p-LRRK2 ^{Ser1292} (1:1000, ab203181, Abcam, U.K.), RAB10 (1:1000, ab181367, Abcam, U.K.), p-RAB10 ^{Thr73} (1:1000, ab231707, Abcam, U.K.), and tubulin (1:10000, E1c601, EnoGene, U.S.A.) overnight at 4°C. The membranes were washed 3 times with Tris-buffered saline/Tween 20 for 5 min, followed by incubation with the peroxidase-conjugated anti-mouse (474-1806) or anti-rabbit (474-1516) IgG (1:5000; KPL) antibody for 1 h at room temperature. The signal was visualized using an ECL western blot detection kit (Millipore, WBKLS0500). ImageJ (National Institutes of Health, Bethesda, Maryland, USA) was used to evaluate the densitometry data. Western blot for tubulin was used as a loading control.

d) RNA extraction and RNA sequencing (RNA-Seq)

Total RNA was isolated from tissues and cell lines using TRIZOL (Invitrogen, 15596-018) according to the manufacturer's instructions. RNA-Seq for mRNA expression profiling of the U251 cells with a stable expression of LRRK2 was performed using the method described in our previous study [41]. In brief, about 1 μg total RNA per sample was used to prepare library for RNA-seq. Sequencing libraries were generated using the NEBNextUltraTM RNA Library Prep kit from Illumina (NEB, USA) following the manufacturer's recommendation and index codes were added to attribute sequence to each sample. The processed final library was sequenced on an IlluminaHiseq 4000 platform, and 150 bp paired ends reads were generated. Raw RNA-seq reads were trimmed to remove sequencing adapters and low-quality reads. The clean reads were then aligned to the reference genome (GRCh38.p7) using HISAT2 [42]. FeatureCounts was used to generate counts for known genes [43]. Gene-level differential expression analyses were performed using DESeq2 [44]. Gene Ontology enrichment analysis of target gene sets was analyzed by DAVID 6.8 (https://david.ncifcrf.gov) [45].

Statistical analysis

Nonparametric statistics (Mann-Whitney) was used to compare the means/distributions of the total PD score and score of each item in the spontaneous PD monkey and the three control monkeys before and after L-dopa or apomorphine treatment. Nonparametric statistics (Mann-Whitney) was

used to compare the means/distributions of the counting data from nigral TH cells, PSer129αS aggregations, microglia cells, and astrocytes. The means/distributions of the TH cells in left and right side SNs were analyzed by the Student's *t*-test. The linear regression between dopamine concentration of CSF samples and the ages of the monkeys was performed by Correlations-double variant & Regression-Curve fit. ANOVA (homogeneous variance) was used to compare the means/distributions of Iba1 labeled microglia's soma diameter. ANOVA with the Tukey's post-hoc test (homogeneous variance) was used to compare the means/distributions of the relative levels of protein expression in Western blot.

Supplementary Results

Identification of the PD risk gene mutations in the Spontaneous PD monkey

It has been well established that PD, especially early-onset PD, has a significant genetic contribution [26, 46, 47]. Because our spontaneous PD monkey represents an early-onset PD case (a 10-year old monkey is approximately equal to a 30-40 years old human being [48, 49]), we subjected the PD monkey and 1,585 cynomolgus monkey controls to a genetic screen by sequencing the coding regions of 11 human PD risk genes (*ATP13A2, FBXO7, GBA, GIGYF2, HTRA2, LRRK2, MAPT, PINK1, PLA2G6, SNCA* and *UCHL1*). In total, 14 missense mutations were found among 6 genes of the spontaneous PD monkey compared to a population of 1,585 cynomolgus monkey controls (Table S3). Subsequently, the identified *LRRK2* and *ATP13A2* rare mutations were verified by Sanger sequencing, and the mutation locations were clearly delineated (Fig. S5).

A sequence alignment of multiple species was conducted to determine if the *LRRK2* mutation sites were conserved during evolution. The alignment indicated that the three *LRRK2* missense mutation sites represented nucleic acid base substitutions in highly conserved loci (Fig. S6) in three functional domains. These included Ankyrin repeats (ANK) that are part of transcription factors and signaling proteins; Ras protein of the GTPase complex (Roc-GTPase), potential regulators of LRRK2 kinase activity, and WD40 domains, the most common repeats in human proteins and found in functionally diverse proteins, such as G protein subunits, protein phosphatase subunits, and RNA processing complexes [50]. Therefore, the identified missense mutations are part of functional domains that may impact *LRRK2* function and promote PD pathogenesis (Fig. S5A). Although the PD monkey *LRRK2* mutations are not the same to humans, their location is close to the ones identified in PD patients [51]. The fact that they are non-synonymous mutations which result in amino acid changes makes it very likely that they affect *LRRK2* functions. In addition, the identified CADD score ≥ 15 is another indicator of a conserved site (Table S3) with a possibly high impact for disease [36, 37]. We also found and verified another missense mutation in the *ATP13A2* gene (Fig. S5B). The ATP13A2 protein is required for normal lysosome function to prevent α-synuclein aggregation in neurons [52, 53].

The sequencing results supported the genetic basis [54, 55], and suggested that a potential dysfunction of *LRRK2* and *ATP13A2* might contribute to the observed spontaneous PD monkey

phenotype. Interestingly, we confirmed that all missense mutations in the spontaneous PD monkey were not present in his available family members including the elder sister (#04106) and father (#95111). Moreover, a behavioral assessment of the elder sister and the father revealed no typical PD symptoms in both animals.

The PD-associated LRRK2 mutations in the spontaneous PD monkey have functional consequences

The next question we asked was whether the *LRRK2* mutations we found in the spontaneous PD monkey can in fact explain PD-related pathology. We focused on the *LRRK2* variants and included the verified *LRRK2* mutant p.G2019S that has been identified in PD patients as a positive control. The three *LRRK2* mutants (p.E2381G, p.N1506Y and p.I835L) identified in the spontaneous PD monkey were generated by using site-directed mutagenesis on the wild type (WT) LRRK2-pCMV-c-Myc vector constructed in our previous study [38] and were verified by sequencing (Table S4). HEK293T cells were then transfected and the lentivirus supernatant produced from HEK293T cells was used to infect U251 cells.

Western blots confirmed stable expression of mutant LRRK2 proteins (Fig. S7A). We then analyzed potential markers relevant for LRRK2 function and PD pathogenesis (Fig. S7B). We found that serine 1292-phosphorylated LRRK2 (p-Ser1292 LRRK2) and threonine 73-phosphorylated Rab10 (p-Thr73 Rab10) levels were both increased (Fig. S7C). We speculate that the increased LRRK2 phosphorylation and Rab10 phosphorylation may contribute to PD pathogenesis of the spontaneous PD monkey. This is based on previous findings that have shown that phosphorylated Rab10 can be activated by LRRK2-mediated phosphorylation to trigger PD pathogenesis [51, 56].

To get a comprehensive understanding regarding the potential role of the LRRK2 mutants, we performed RNA sequencing (RNA-Seq) and found that many genes were differentially expressed in *LRRK2* mutant overexpressing cells compared to controls (positive control p.G2019S: 26 genes; p.N1506Y: 142 genes; p.E2381G: 58 genes, and p.I835L: 663 genes) (Fig. S7D). Affected pathways of three *LRRK2* mutants were related to immunologic dysfunction (Fig. S7F-H), which is one of the current mainstream hypothesis of PD pathogenesis. Collectively, the RNA-Seq analysis data indicated that activated immunologic reactions or inflammations may contribute to PD pathogenesis

in the spontaneous PD monkey, which is also consistent with the glial cell activation we observed.

To further confirm the pathological changes induced by the *LRRK2* mutants in U251 cultured cells, we selected PSer129αS as a pathology marker for immunofluorescence staining. We found that PSer129αS is expressed and accumulated at high levels in all *LRRK2* mutant cells compared with wild type and PLVX control cells and with most of the protein located in the cytoplasm (Fig. S8). This finding was consistent with the α-synuclein pathology that we had found in the spontaneous PD monkey.

The functional tests *in vitro* suggested that the *LRRK2* mutations we found in the spontaneous PD monkey can at least partially explain its PD-related pathology.

Supplementary Figures

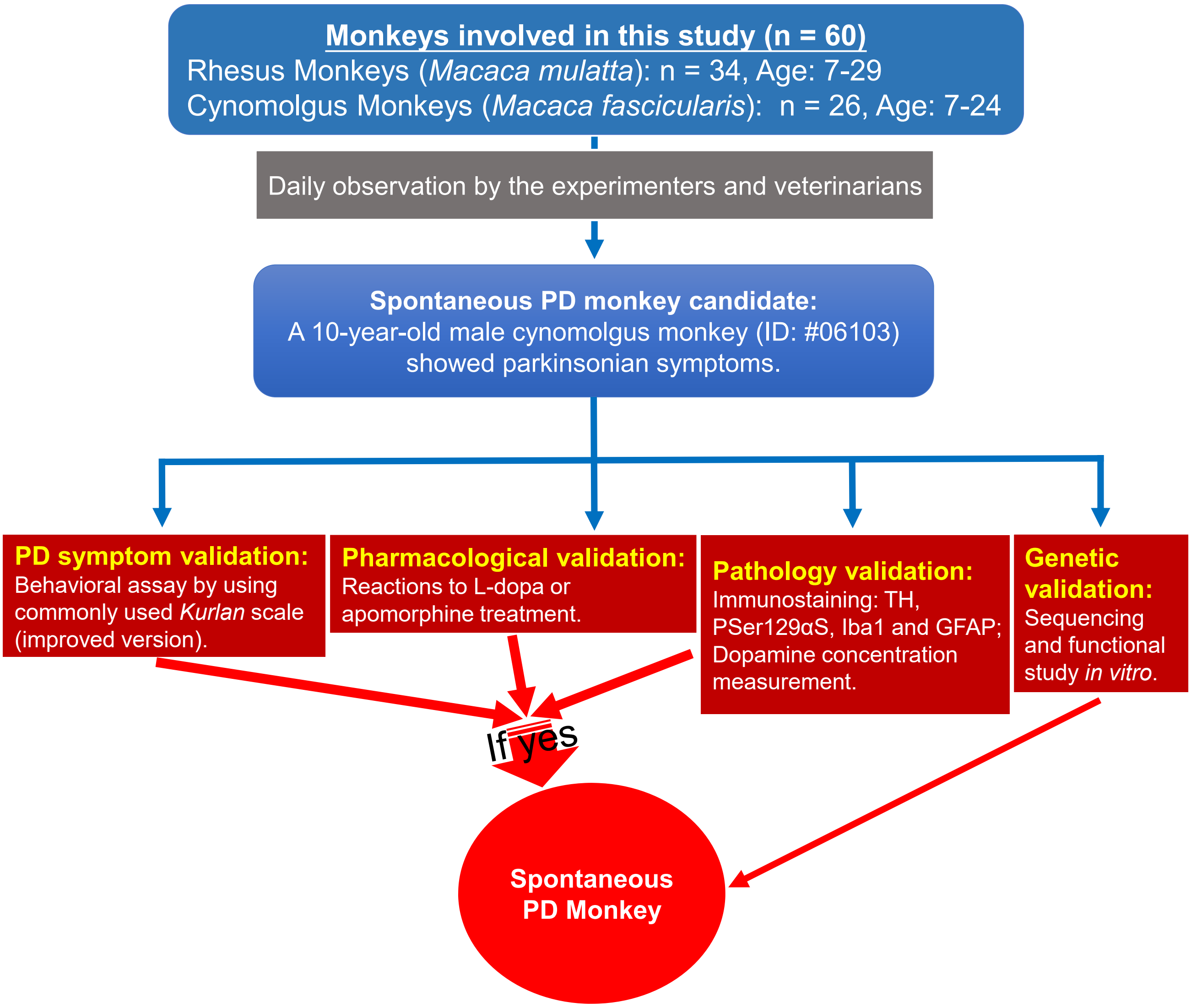

Figure S1. A flow chart shows the search process (blue and gray panel) and four validation steps (red panels) used in the screening of spontaneous PD monkey candidates.

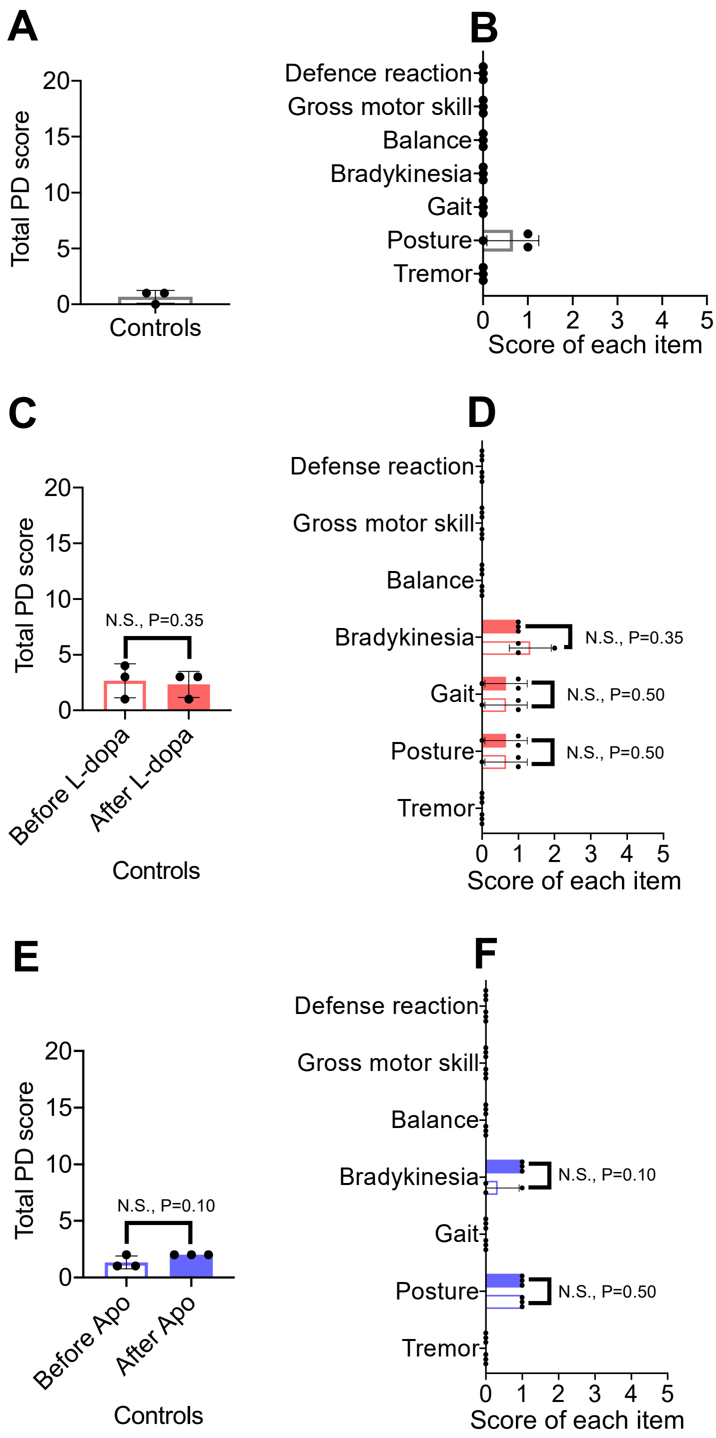

Figure S2. PD symptom assessment of the three control monkeys. (**A**) Quantified by the improved *Kurlan* scale, the averaged total PD score of the controls was around 1, which was within the normal range according to the scale (0 to 4). (**B**) Seven items that make up the scale were marked zero except for the posture, which was less than 1. (**C**) The averaged total PD score of the controls was not significantly changed after the L-dopa treatment. (**D**) The seven items were not significantly changed after the L-dopa treatment. (**E**) The averaged total PD score of the controls was not significantly changed after the Apo treatment. (**F**) The seven items were not significantly changed after the Apo treatment. Data are means ± SD. Nonparametric statistics (Mann-Whitney test) was used. N.S., not significant.

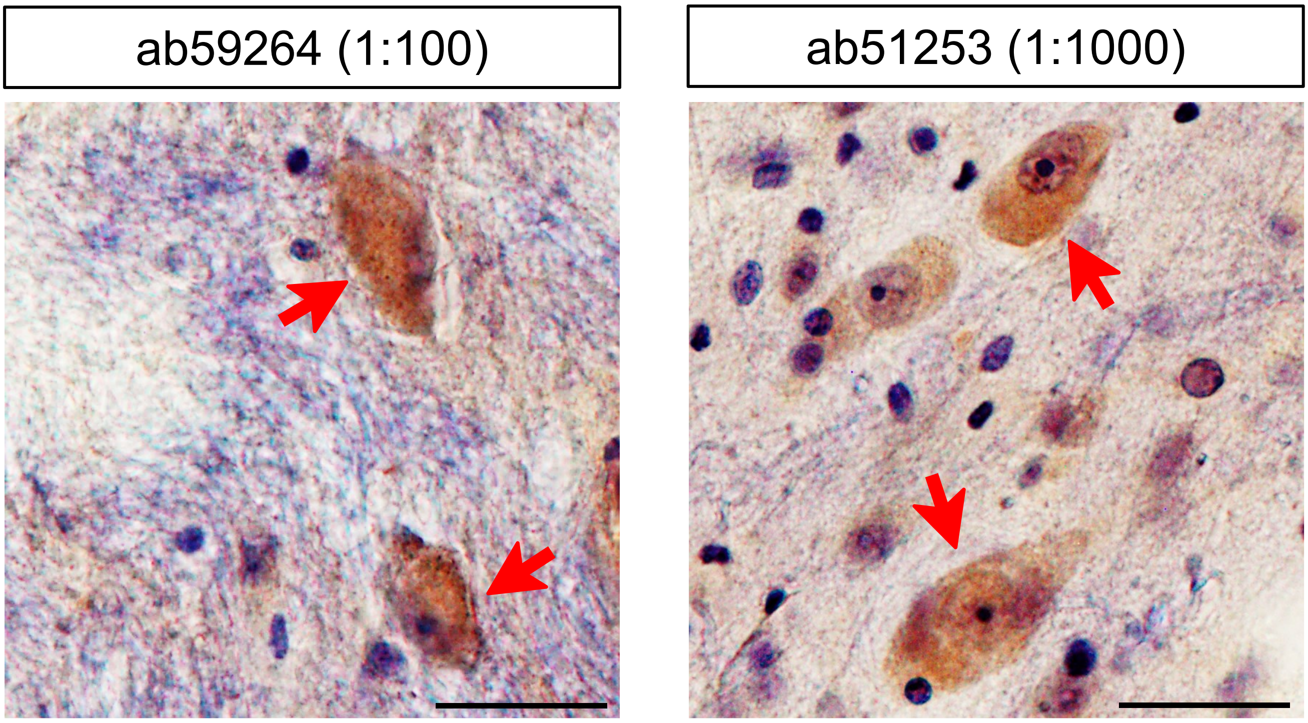

Figure S3. Comparison of two antibodies staining of phosphorylated-Ser129 α-synuclein (PSer129αS) in locus ceruleus (LC) of the spontaneous PD monkey. The rabbit polyclonal antibody (ab59264, 1: 100) and the rabbit monoclonal antibody (ab51253, 1: 1000) can both stain the PSer129αS aggregates (red arrows). The results obtained from both are similar. Scale bar: 20 μm.

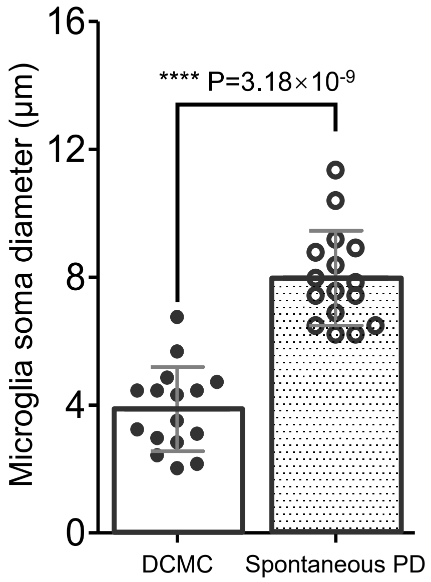

Figure S4. The average soma diameters of microglia in the spontaneous PD monkey (ID: #06103) were significantly larger than that of the death-condition-matched control (DCMC, ID: #071809). For each monkey, one section (section #34) was picked. The location of the counting frame under a 40 × objective was similar to that of nigral dopaminergic neurons quantification, from which 16 typical $Iba1^+$ cells were selected for measuring the soma diameter by ImageJ software. Data are means ± SD. One-way ANOVA was used for the statistics.

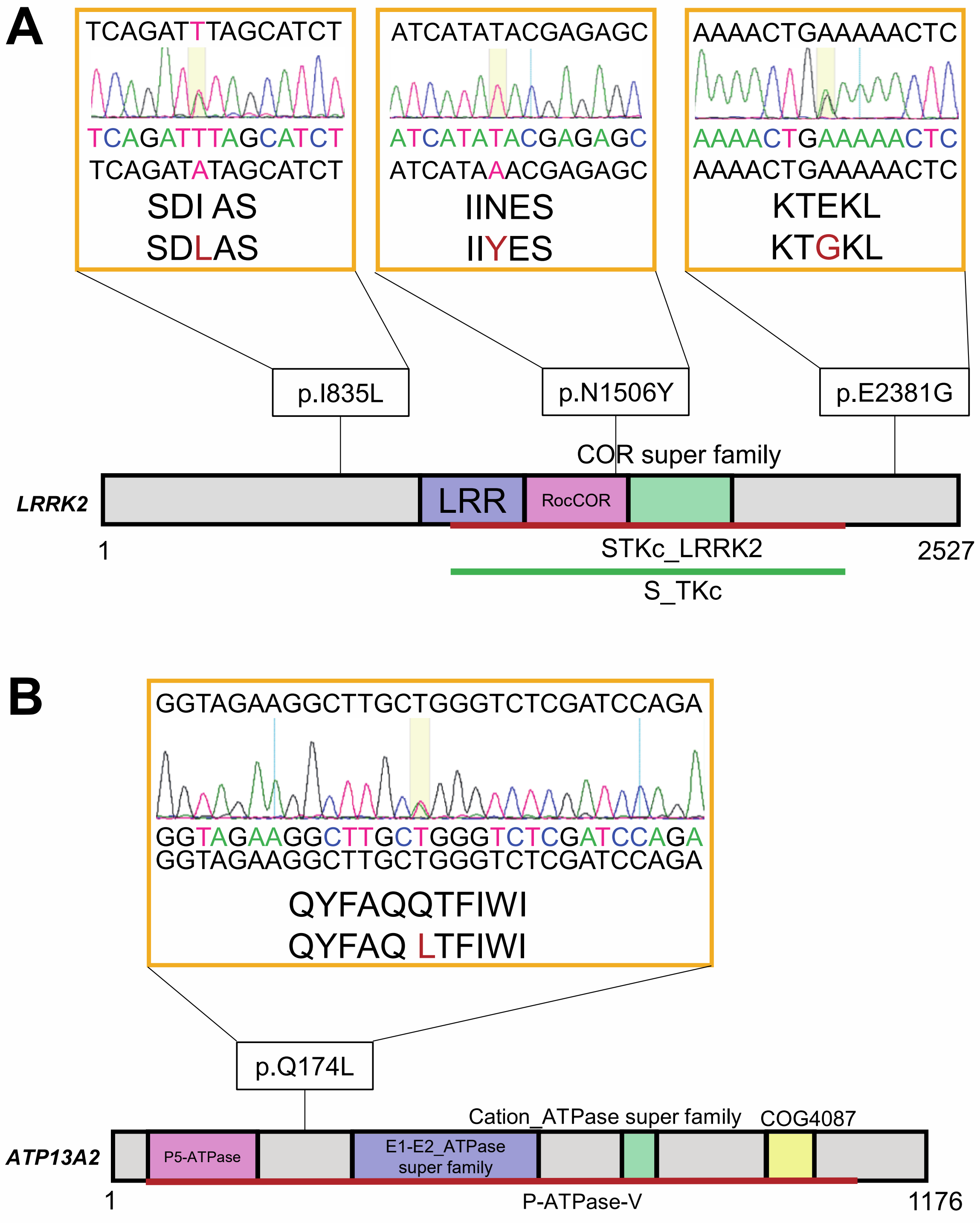

Figure S5. The LRRK2 and ATP13A2 missense mutations of the spontaneous PD monkey. (**A**) Sequencing verification of the LRRK2 mutations. (**B**) Sequencing verification of the ATP13A2 mutation.

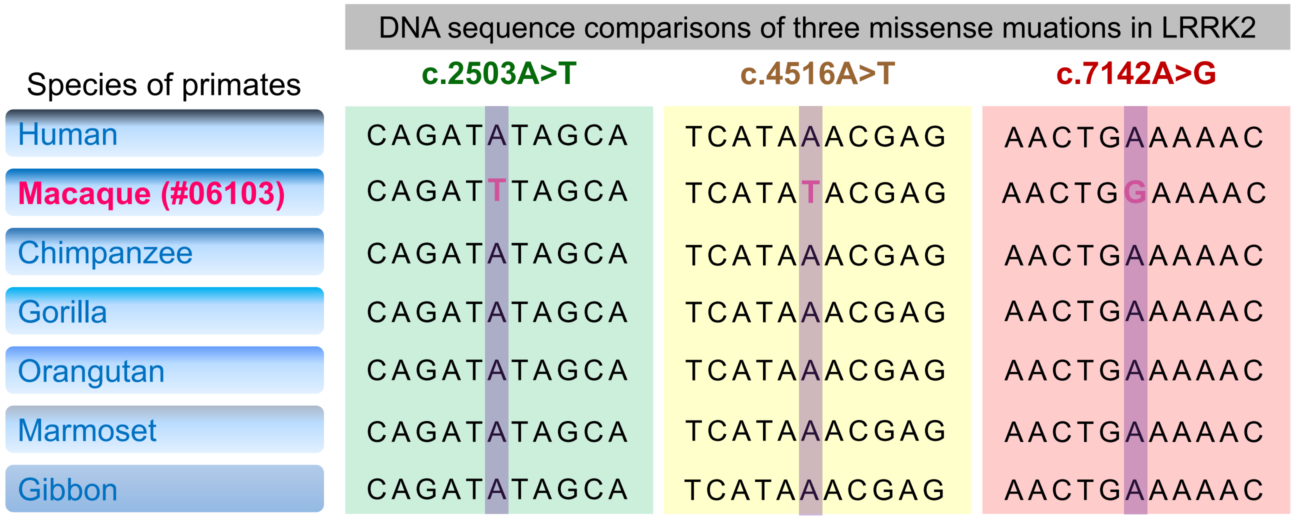

Figure S6. Alignment of primates *LRRK2* gene sequences. The missense mutations of LRRK2 identified in the spontaneous PD monkey (ID: #06103) were compared with 6 other primate species.

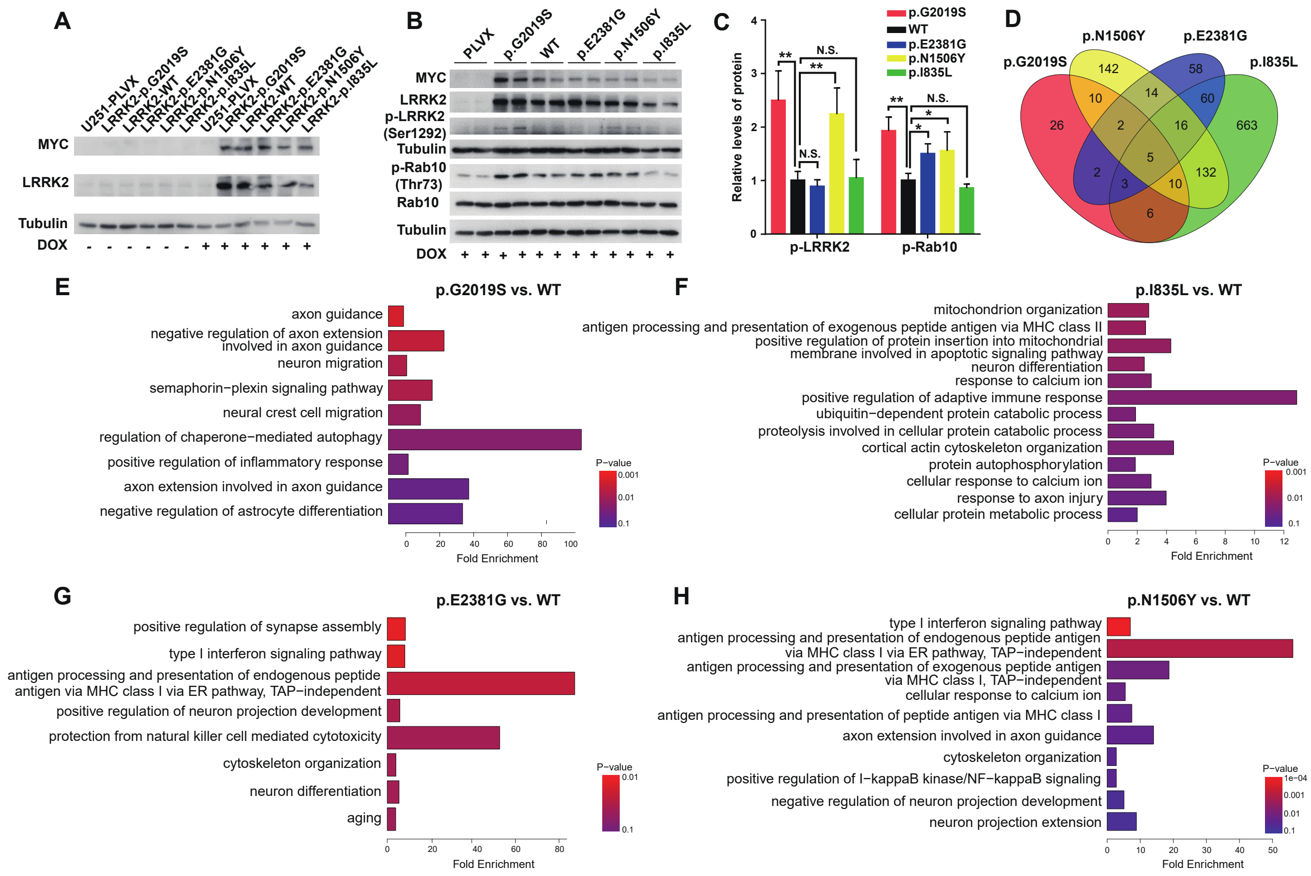

Figure S7. Functional characterization of the LRRK2 mutants identified in the spontaneous PD monkey. (**A**) Immunoblot analysis of total U251 cell lysates with stable expression of wild type (WT) and mutant LRRK2 induced by doxycycline (2 μg/mL) treatment. (**B**) Protein levels of LRRK2, p-LRRK2, p-Rab10, and Rab10 in U251 cell lysates with stable expression of wild type and mutant LRRK2 in the presence of doxycycline. (**C**) Densitometry of the proteins in figure (**B**). Relative protein abundance was normalized to tubulin. Data is representative of 3 independent experiments with similar results. *, $P<0.05$; **, $P<0.01$; N.S., not significant. One-way ANOVA with the Tukey's post-hoc test was used. Data in figure (**C**) are means ± SD. (**D-H**) Differentially expressed genes (DEGs) and disrupted pathways of DEGs in U251 cells with stable expression of different LRRK2 mutants induced by doxycycline (2 μg/mL) treatment. (**D**) Total number of differentially expressed genes. (**E-H**) Enriched pathways in U251 cells with stable expression of LRRK2 mutations p.G2019S (**E**), p.I835L (**F**), p.E2381G (**G**), and p.N1506Y (**H**), relative to wild type (WT) LRRK2. Enriched biological processes in Gene Ontology (GO) of the target gene sets were identified by DAVID 6.8 (https://david.ncifcrf.gov) [45]. Shown are enriched terms with $P<0.05$; x-axis, fold enrichment; y-axis, enriched GO terms.

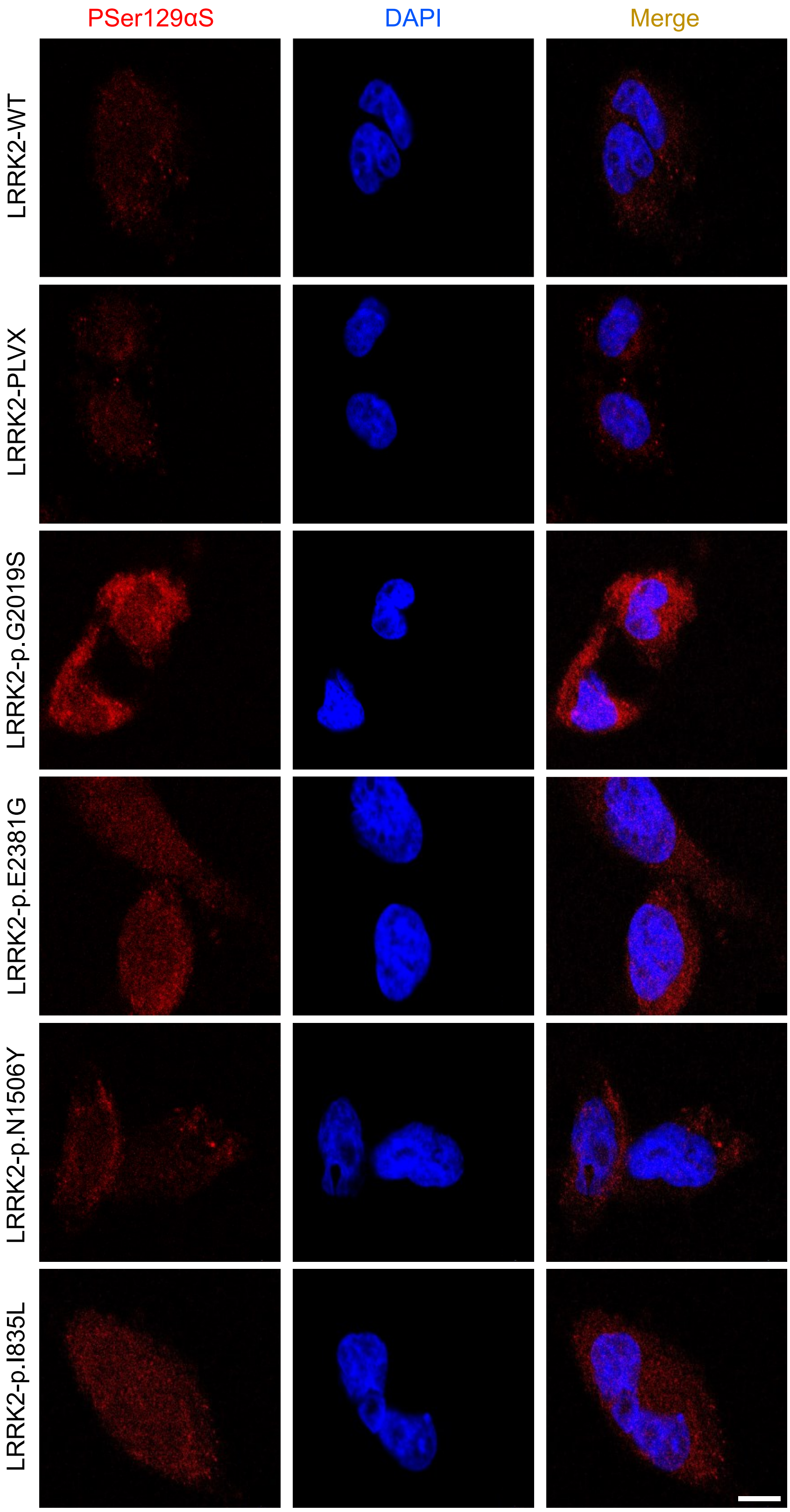

Figure S8. Effect of different LRRK2 mutants on the formation of intracellular PSer129αS aggregates in U251 cells using immunofluorescence. Data are representative of three independent experiments with similar results. Scale bar: 10 μm.

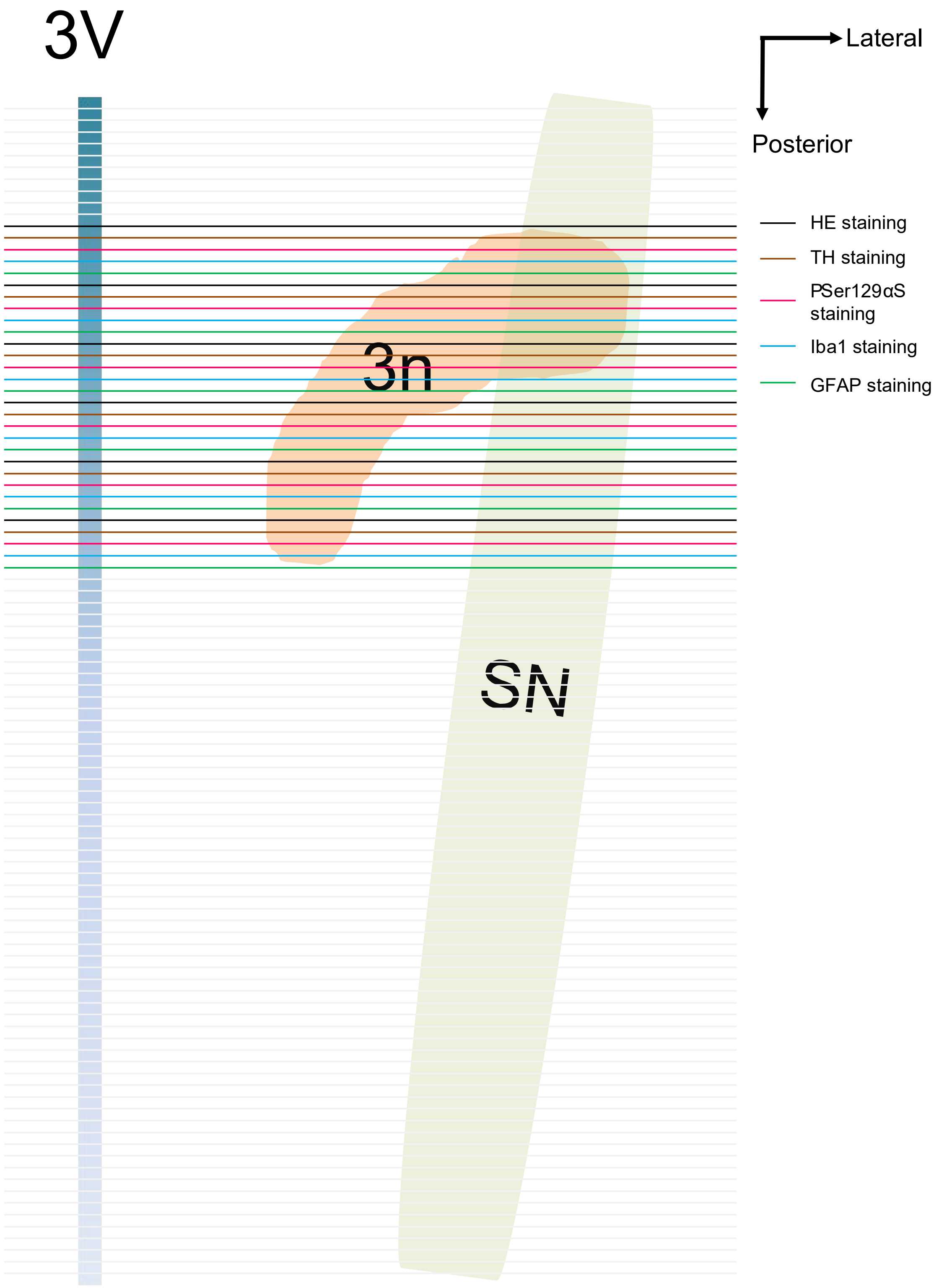

Figure S9 Diagram illustrating how the 30 optimal sections of a monkey's right substantia nigra (SN) were divided into six sets for HE, TH, P-ser 129 α-Syn, Iba1 and GFAP staining, respectively. Each set has 5 sections, which are marked with five lines in specific colors. A monkey's SN was sectioned into 100 sections with an interval of 60 μm in between. Each line represents one section within a thickness of 20 μm. The 30 sections overlapped with the 3n are optimum for nigral dopaminergic cell counting [9]. 3n: the third nerve, 3V: the third ventricle.

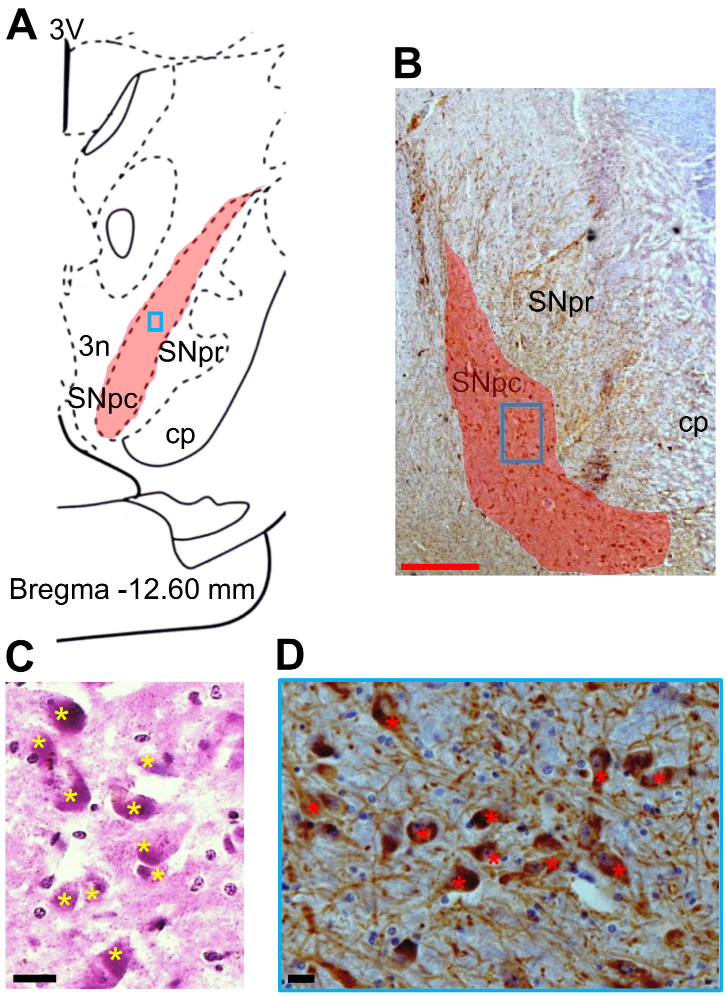

Figure S10. Based on the SNpc anatomical and dopaminergic neuron morphological features, the location of a counting frame for stereological counting in a substantia nigra (SN) section of the death-condition-matched control (DCMC) was determined. (**A**) A diagram of a monkey's SN and related structures modified from "*The Rhesus Monkey Brain in Stereotaxic Coordinates, George Praxinos et al., Academic Press, 1999*". The red shaded area shows the whole unilateral SNpc and the location of a blue counting frame, which is in the middle of the long axis of the SNpc. The area inside the blue frame is for the counting of dopaminergic neurons. (**B**) A nigral TH immunostaining image of the DCMC under a 4 × objective (3.5 mm × 2.6 mm). The red shaded area shows the whole unilateral SNpc and the blue counting frame in the middle of the SNpc shows the area for cell counting. (**C**) Nigral pigmented cells marked with yellow asterisks in a H & E image under a 40 × objective (345.4 μm × 259 μm). (**D**) Spindle-shaped nigral dopaminergic neurons marked with red asterisks in a TH immunostaining image under a 40 × objective. This is the area in the blue frame of figure (**B**). Both (**C**) and (**D**) show the dopaminergic neuron morphological features for determining the location of the counting frame in the SNpc: clear and evenly distributed dopaminergic neurons in the area, which is indicated by both HE staining and TH immunostaining in (**C**) and (**D**), respectively. After careful comparison, we assumed the location of the blue counting frame should be in the middle of the long axis of the SNpc, and this location was applied to all the 30 optimum sections. Scale bars: red: 500 μm, black: 20 μm. 3n: the third cranial nerve; 3V: the third ventricle; cp: the cerebral peduncle; SNpc: substantia nigra pars compacta; SNpr: substantia nigra pars reticulata.

Supplementary Tables

Table S1. Detailed information of the 60 monkeys involved in the study. The 60 monkeys were selected from the colonies (2,400+ individuals) at the Kunming Primate Research Center, Kunming Institute of Zoology, Chinese Academy of Sciences. The spontaneous PD monkey (ID: #06103) is labeled in red. A death-condition-matched control (DCMC, ID: #071809) is labeled in gray, and two age-matched control monkeys (ID: #071725 & #05103) are labeled in blue. The eight monkeys labeled in green were normal controls for CSF dopamine concentration test. *indicates age of each monkey that was part of the study.

Rhesus monkey (n = 34)			Cynomolgus monkey (n = 26)		
**ID: #**	**Age***	**Gender**	**ID: #**	**Age***	**Gender**
070141	9	Male	06101	10	Male
060113	10	Male	06103	10	Male
090019	7	Male	95111	24	Male
705351	9	Male	04106	14	Female
070209	9	Male	080011	8	Male
070155	9	Male	071725	9	Male
070229	9	Male	05103	11	Male
080227	8	Male	071809	9	Male
070179	9	Male	080009	8	Male
06411	10	Male	08103	8	Male
06415	10	Male	071023	9	Male
07451	9	Male	071905	9	Male
08005	8	Male	080015	8	Male
08031	8	Male	071069	9	Male
08301	8	Male	08101	10	Male
08377	8	Male	00107	18	Male
08419	8	Male	04107	14	Male
91327	25	Male	04109	14	Male
93334	23	Female	060245	12	Male
94354	22	Female	11133	7	Male
94356	22	Female	08105	8	Male
89323	27	Male	97101	21	Male
93332	23	Female	09103	9	Male
89313	27	Male	11137	7	Male
91319	25	Male	051090	11	Female
90309	26	Male	060309	12	Male
92348	24	Female			
92350	24	Female			
92330	24	Female			
91313	25	Male			
89325	27	Male			
90319	26	Male			
94338	22	Female			
87033	29	Male			

Table S2. Improved version of the *Kurlan* scale (*A Monkey-Parkinsonism Rating Scale*). The part A and part D are listed.

Part A. Parkinsonian features (20 scores in total)

1.Tremor (L/R): like the tremor of PD patients
- **0**-absent
- **1**-slight-low amplitude and only intermittently present
- **2**-moderate-moderate amplitude and present most of the time
- **3**-severe-high amplitude, virtually continuous, interferes with function

2.Posture: different from the postural instability of PD patients, not applicable to PD patients
- **0**-normal, erect
- **1**-stooped
- **2**-face down

3.Gait: partly like the postural instability of PD patients
- **0**-normal, use all four limbs smoothly
- **1**-walks slowly
- **2**-marked impaired, able to ambulate but very slowly and with effort
- **3**-severe decrease in ability to ambulate
- **4**-unable to ambulate

4.Bradykinesia (generalized): like the bradykinesia of PD patients
- **0**-normal speed and facility of movement
- **1**-mild slowing of overall movements
- **2**-moderate slowing of movements
- **3**-severe slowing of movements: slow, labored, and difficult to initiate and maintain movement
- **4**-unable to ambulate

5.Balance: partly like the postural instability of PD patients
- **0**-normal balance
- **1**-mild loss of balance on arising or with movement, holds onto cage for support
- **2**-major lapse in balance

6.Gross motor skills (upper limb, L/R): partly like the postural instability of PD patients
- **0**-normal, uses limb through a wide range of motion and activities
- **1**-noticeable decrease in capacity to use limb, but used consistently
- **2**-severe decrease in capacity to use limb, rarely used
- **3**-unable or refuses to use limb (including walking)

7.Defense reaction (defensive and/or aggressive response to examiner): not applicable to PD patients
- **0**-normal, reacts appropriately
- **1**-detectable impaired, slowed, abnormal or shortened response
- **2**-little or no response upon good provocation

Part D. Clinical staging (5 stages)
- **I.** Hemi-Parkinsonism, uses affected upper limb for walking
- **II.** Hemi-Parkinsonism, does not use affected upper limb for walking
- **III.** Bilateral Parkinsonism, maintains adequate nutrition without assistance
- **IV.** Bilateral Parkinsonism, requires assistance to maintain adequate nutrition, maintains upright position
- **V.** Bilateral Parkinsonism, requires assistance to maintain adequate nutrition, face-down position

Note:
Part A was used to determine the Parkinsonian score while part D provided clinical measures of the Parkinsonian stage [2].

Table S3. MIP sequencing information for PD-risk / causal genes in the spontaneous PD monkey (ID: #06103). Combined Annotation Dependent Depletion (CADD) was used as a tool for scoring the effect size of single nucleotide variants [36, 37]. A CADD score ≥ 15 is an indicator of conserved sites, which could possibly have a high impact on PD pathogenesis.

Chromosome	Position	Reference Base	Sample Genotype	Sample Alleles	Function GVS	Amino Acids	Protein Position	cDNA Position	Chimp Allele	Gene List	Protein Sequence	CADD Scores
12	40704431	A	T	T/T	missense	ASN, TYR	1506/2528	4516	A	LRRK2	NP_940980.3	21.3
22	38508211	C	T	T/T	missense	ARG, HIS	739/753	2216	C	PLA2G6	NP_001004426.1	12.54
2	233655834	T	G	G/G	missense	ASP, GLU	349/1300	1047	T	GIGYF2	NP_001096616.1	10.43
1	155209708	C	T	T/T	missense	MET, ILE	92/537	276	C	GBA	NP_000148.2	7.153
1	17328861	C	T	T/T	missense	ASP, ASN	184/1176	550	C	ATP13A2	NP_001135445.1	18.21
22	38541638	C	Y	C/T	missense	ALA, THR	78/753	232	C	PLA2G6	NP_001004426.1	10.25
12	40757317	A	R	A/G	missense	GLU, GLY	2381/2528	7142	A	LRRK2	NP_940980.3	20.8
1	17316247	C	Y	C/T	missense	VAL, ILE	845/1176	2533	C	ATP13A2	NP_001135445.1	11.55
4	41259691	G	S	C/G	missense	GLU, ASP	37/224	111	G	UCHL1	NP_004172.2	12.39
1	17330848	T	W	A/T	missense	GLN, LEU	174/1176	521	T	ATP13A2	NP_001135445.1	12.44
1	17331258	C	Y	C/T	missense	ALA, THR	136/1176	406	C	ATP13A2	NP_001135445.1	10.28
1	155209520	A	R	A/G	missense	PHE, SER	114/537	341	A	GBA	NP_000148.2	11.54
1	155209489	C	Y	C/T	missense	MET, ILE	124/537	372	C	GBA	NP_000148.2	14.03
12	40681155	A	W	A/T	missense	ILE, LEU	835/2528	2503	A	LRRK2	NP_940980.3	8.162

Table S4. Primers for cloning and site mutagenesis of the *LRRK2* gene.

Primer [a]	Sequence (5'-3')	Application
**For site mutation**		
p.E2381G-F	GGGATAAGAAAACT***G***GAAAACTCTGTGGAC	Mutant E2381G-LRRK2
p.E2381G-R	GTCCACAGAGTTTTC***C***AGTTTTCTTATCCC	
p.N1506Y-F	CGGAAAACCATCA***T***ATACGAGAGCCTTAAT	Mutant N1506Y-LRRK2
p.N1506Y-R	ATTAAGGCTCTCGTAT***A***TGATGGTTTTCCG	
p.I835L-F	AAGGAAACAAACAAATT***T***AGCATCTACACTAG	Mutant I835L-LRRK2
p.I835L-R	CTAGTGTAGATGCT***A***AATTTGTTTGTTTCCTT	
**For lentivirus construct [b]**		
NotI-PLVX-F	ATAAGAAT***GCGGCCGC***ATGGCATCAATGCAGAAG	Forward primer for vector construction
MluI-PLVX-R	CG***ACGCGT***TTACTCAACAGATGTTCGTCTC	Reverse primer for vector construction

[a] Restriction recognition sites are indicated with italic, bold and underlining fonts directed mutagenesis sites are highlighted in red. F-forward primer; R-reverse primer. [b] Primers used in our previous study [38].

Supplementary References

1. Kilkenny, C, Browne, WJ, Cuthill, IC*, et al.* Improving bioscience research reporting: the ARRIVE guidelines for reporting animal research. *PLoS Biol*. 2010; **8**(6): e1000412.
2. Smith, RD, Zhang, Z, Kurlan, R*, et al.* Developing a stable bilateral model of parkinsonism in rhesus monkeys. *Neuroscience*. 1993; **52**(1): 7-16.
3. Imbert, C, Bezard, E, Guitraud, S*, et al.* Comparison of eight clinical rating scales used for the assessment of MPTP-induced parkinsonism in the Macaque monkey. *J Neurosci Methods*. 2000; **96**(1): 71-6.
4. Lei, X, Li, H, Huang, B*, et al.* 1-methyl-4-phenylpyridinium stereotactic infusion completely and specifically ablated the nigrostriatal dopaminergic pathway in rhesus macaque. *PLoS One*. 2015; **10**(5): e0127953.
5. Li, H, Lei, X, Huang, B*, et al.* A quantitative approach to developing Parkinsonian monkeys (Macaca fascicularis) with intracerebroventricular 1-methyl-4-phenylpyridinium injections. *J Neurosci Methods*. 2015; **251**: 99-107.
6. Bezard, E, Przedborski, S. A tale on animal models of Parkinson's disease. *Mov Disord*. 2011; **26**(6): 993-1002.
7. Beal, MF. Experimental models of Parkinson's disease. *Nature reviews Neuroscience*. 2001; **2**(5): 325-34.
8. Emborg, ME. Nonhuman primate models of Parkinson's disease. *ILAR J*. 2007; **48**(4): 339-55.
9. Dickson, DW, Braak, H, Duda, JE*, et al.* Neuropathological assessment of Parkinson's disease: refining the diagnostic criteria. *Lancet Neurol*. 2009; **8**(12): 1150-7.
10. Chu, Y, Kompoliti, K, Cochran, EJ*, et al.* Age-related decreases in Nurr1 immunoreactivity in the human substantia nigra. *J Comp Neurol*. 2002; **450**(3): 203-14.
11. Chu, Y, Kordower, JH. Age-associated increases of alpha-synuclein in monkeys and humans are associated with nigrostriatal dopamine depletion: Is this the target for Parkinson's disease? *Neurobiol Dis*. 2007; **25**(1): 134-49.
12. Chu, Y, Muller, S, Tavares, A*, et al.* Intrastriatal alpha-synuclein fibrils in monkeys: spreading, imaging and neuropathological changes. *Brain*. 2019.
13. Gundersen, HJ, Bagger, P, Bendtsen, TF*, et al.* The new stereological tools: disector, fractionator, nucleator and point sampled intercepts and their use in pathological research and diagnosis. *APMIS*. 1988; **96**(10): 857-81.
14. Huang, B, Wu, S, Wang, Z*, et al.* Phosphorylated alpha-Synuclein Accumulations and Lewy Body-like Pathology Distributed in Parkinson's Disease-Related Brain Areas of Aged Rhesus Monkeys Treated with MPTP. *Neuroscience*. 2018; **379**: 302-15.
15. Neumann, M, Kahle, PJ, Giasson, BI*, et al.* Misfolded proteinase K-resistant hyperphosphorylated alpha-synuclein in aged transgenic mice with locomotor deterioration and in human alpha-synucleinopathies. *J Clin Invest*. 2002; **110**(10): 1429-39.
16. Shahmoradian, SH, Lewis, AJ, Genoud, C*, et al.* Lewy pathology in Parkinson's disease consists of crowded organelles and lipid membranes. *Nat Neurosci*. 2019; **22**(7): 1099-109.
17. Bae, EJ, Yang, NY, Song, M*, et al.* Glucocerebrosidase depletion enhances cell-to-cell transmission of alpha-synuclein. *Nat Commun*. 2014; **5**: 4755.
18. Gan, L, Vargas, MR, Johnson, DA*, et al.* Astrocyte-specific overexpression of Nrf2 delays motor pathology and synuclein aggregation throughout the CNS in the alpha-synuclein mutant (A53T) mouse model. *J Neurosci*. 2012; **32**(49): 17775-87.
19. Kiely, AP, Asi, YT, Kara, E*, et al.* alpha-Synucleinopathy associated with G51D SNCA mutation: a link between Parkinson's disease and multiple system atrophy? *Acta Neuropathol*. 2013; **125**(5): 753-69.
20. Ling, H, Kara, E, Bandopadhyay, R*, et al.* TDP-43 pathology in a patient carrying G2019S LRRK2 mutation and a novel p.Q124E MAPT. *Neurobiol Aging*. 2013; **34**(12): 2889 e5-9.
21. Mestre-Frances, N, Serratrice, N, Gennetier, A*, et al.* Exogenous LRRK2G2019S induces parkinsonian-like pathology in a nonhuman primate. *JCI Insight*. 2018; **3**(14).
22. Lohmann, S, Bernis, ME, Tachu, BJ*, et al.* Oral and intravenous transmission of alpha-synuclein fibrils to mice. *Acta Neuropathol*. 2019; **138**(4): 515-33.
23. Garcia-Dominguez, I, Vesela, K, Garcia-Revilla, J*, et al.* Peripheral Inflammation Enhances Microglia Response and Nigral Dopaminergic Cell Death in an in vivo MPTP Model of Parkinson's Disease. *Front Cell Neurosci*. 2018; **12**: 398.
24. Kimura, K, Inoue, KI, Kuroiwa, Y*, et al.* Propagated but Topologically Distributed Forebrain Neurons Expressing Alpha-Synuclein in Aged Macaques. *PLoS One*. 2016; **11**(11): e0166861.
25. Wegrzynowicz, M, Bar-On, D, Calo, L*, et al.* Depopulation of dense alpha-synuclein aggregates is associated with rescue of dopamine neuron dysfunction and death in a new Parkinson's disease model. *Acta Neuropathol*. 2019; **138**(4): 575-95.

26. Poewe, W, Seppi, K, Tanner, CM*, et al.* Parkinson disease. *Nat Rev Dis Primers*. 2017; **3**: 17013.
27. Kraemmer, J, Kovacs, GG, Perju-Dumbrava, L*, et al.* Correlation of striatal dopamine transporter imaging with post mortem substantia nigra cell counts. *Mov Disord*. 2014; **29**(14): 1767-73.
28. Houlden, H, Singleton, AB. The genetics and neuropathology of Parkinson's disease. *Acta Neuropathol*. 2012; **124**(3): 325-38.
29. Singleton, AB, Farrer, MJ, Bonifati, V. The genetics of Parkinson's disease: progress and therapeutic implications. *Mov Disord*. 2013; **28**(1): 14-23.
30. Boyle, EA, O'Roak, BJ, Martin, BK*, et al.* MIPgen: optimized modeling and design of molecular inversion probes for targeted resequencing. *Bioinformatics*. 2014; **30**(18): 2670-2.
31. Hardenbol, P, Baner, J, Jain, M*, et al.* Multiplexed genotyping with sequence-tagged molecular inversion probes. *Nat Biotechnol*. 2003; **21**(6): 673-8.
32. Nilsson, M, Malmgren, H, Samiotaki, M*, et al.* Padlock probes: circularizing oligonucleotides for localized DNA detection. *Science*. 1994; **265**(5181): 2085-8.
33. O'Roak, BJ, Vives, L, Fu, W*, et al.* Multiplex targeted sequencing identifies recurrently mutated genes in autism spectrum disorders. *Science*. 2012; **338**(6114): 1619-22.
34. Wang, T, Guo, H, Xiong, B*, et al.* De novo genic mutations among a Chinese autism spectrum disorder cohort. *Nat Commun*. 2016; **7**: 13316.
35. Li, H, Durbin, R. Fast and accurate long-read alignment with Burrows-Wheeler transform. *Bioinformatics*. 2010; **26**(5): 589-95.
36. Kircher, M, Witten, DM, Jain, P*, et al.* A general framework for estimating the relative pathogenicity of human genetic variants. *Nat Genet*. 2014; **46**(3): 310-5.
37. Rentzsch, P, Witten, D, Cooper, GM*, et al.* CADD: predicting the deleteriousness of variants throughout the human genome. *Nucleic Acids Res*. 2019; **47**(D1): D886-D94.
38. Wang, D, Xu, L, Lv, L*, et al.* Association of the LRRK2 genetic polymorphisms with leprosy in Han Chinese from Southwest China. *Genes Immun*. 2015; **16**(2): 112-9.
39. Su, LY, Li, H, Lv, L*, et al.* Melatonin attenuates MPTP-induced neurotoxicity via preventing CDK5-mediated autophagy and SNCA/alpha-synuclein aggregation. *Autophagy*. 2015; **11**(10): 1745-59.
40. Su, LY, Luo, R, Liu, Q*, et al.* Atg5- and Atg7-dependent autophagy in dopaminergic neurons regulates cellular and behavioral responses to morphine. *Autophagy*. 2017; **13**(9): 1496-511.
41. Xu, M, Zhang, DF, Luo, R*, et al.* A systematic integrated analysis of brain expression profiles reveals YAP1 and other prioritized hub genes as important upstream regulators in Alzheimer's disease. *Alzheimers Dement*. 2018; **14**(2): 215-29.
42. Kim, D, Langmead, B, Salzberg, SL. HISAT: a fast spliced aligner with low memory requirements. *Nat Methods*. 2015; **12**(4): 357-60.
43. Liao, Y, Smyth, GK, Shi, W. featureCounts: an efficient general purpose program for assigning sequence reads to genomic features. *Bioinformatics*. 2014; **30**(7): 923-30.
44. Love, MI, Huber, W, Anders, S. Moderated estimation of fold change and dispersion for RNA-seq data with DESeq2. *Genome Biol*. 2014; **15**(12): 550.
45. Huang da, W, Sherman, BT, Lempicki, RA. Systematic and integrative analysis of large gene lists using DAVID bioinformatics resources. *Nat Protoc*. 2009; **4**(1): 44-57.
46. Kalia, LV, Lang, AE. Parkinson's disease. *Lancet*. 2015; **386**(9996): 896-912.
47. Lees, AJ, Hardy, J, Revesz, T. Parkinson's disease. *Lancet*. 2009; **373**(9680): 2055-66.
48. Roth, GS, Mattison, JA, Ottinger, MA*, et al.* Aging in rhesus monkeys: relevance to human health interventions. *Science*. 2004; **305**(5689): 1423-6.
49. Tigges, J, Gordon, TP, McClure, HM*, et al.* Survival rate and life span of rhesus monkeys at the Yerkes regional primate research center. *Am J Primatol*. 1988; **15**(3): 263-73.
50. Yu, L, Gaitatzes, C, Neer, E*, et al.* Thirty-plus functional families from a single motif. *Protein Sci*. 2000; **9**(12): 2470-6.
51. Mata, IF, Wedemeyer, WJ, Farrer, MJ*, et al.* LRRK2 in Parkinson's disease: protein domains and functional insights. *Trends Neurosci*. 2006; **29**(5): 286-93.
52. Ramirez, A, Heimbach, A, Grundemann, J*, et al.* Hereditary parkinsonism with dementia is caused by mutations in ATP13A2, encoding a lysosomal type 5 P-type ATPase. *Nat Genet*. 2006; **38**(10): 1184-91.
53. Yang, X, Xu, Y. Mutations in the ATP13A2 gene and Parkinsonism: a preliminary review. *Biomed Res Int*. 2014; **2014**: 371256.
54. Cookson, MR. The role of leucine-rich repeat kinase 2 (LRRK2) in Parkinson's disease. *Nature reviews Neuroscience*. 2010; **11**(12): 791-7.
55. Xu, Q, Guo, H, Zhang, X*, et al.* Hypoxia regulation of ATP13A2 (PARK9) gene transcription. *J Neurochem*. 2012; **122**(2): 251-9.
56. Eyers, PA. Back to the future: new target-validated Rab antibodies for evaluating LRRK2 signalling in cell biology and Parkinson's disease. *Biochem J*. 2018; **475**(1): 185-9.
